# Do Measures of Systemizing and Empathizing Reflect Perceptions of Gender Differences in Learning Affordances?

**DOI:** 10.1177/01461672231202268

**Published:** 2023-10-21

**Authors:** Audrey Aday, Toni Schmader, Michelle Ryan

**Affiliations:** 1 The University of British Columbia, Vancouver, Canada; 2 The Australian National University, Canberra, Australian Capital Territory, Australia

**Keywords:** systemizing, empathizing, sex/gender differences, affordances

## Abstract

Gender differences in systemizing and empathizing are sometimes attributed to inherent biological factors. We tested whether such effects are more often interpreted as reflecting men’s and women’s different learning affordances. Study 1 (*N =* 624) estimated gender differences in item-level activities from systemizing and empathizing scales (SQ, EQ) in large representative samples. Lay coders (Study 2, *N =* 199) and psychology experts (Study 3, *N =* 116) rated SQ and EQ activities as being more learned (vs. innate) and believed that men receive more systemizing and women receive more empathizing (Study 3 only) affordances. Items showing the largest gender differences in Study 1 were those rated as having the largest gender affordances (more than gendered genetic advantages) in Studies 2 and 3. Claims about inherent sex differences in systemizing, and to a lesser degree empathizing, appear to be out of step with a consensus view from the public and psychological scientists.

In 2017, Google engineer James Damore disseminated an internal memo where he asserted, “On average, men and women biologically differ in many ways [. . . that] may explain why we don’t see equal representation of women in tech and leadership” (p. 3). He cited women’s lower interest in *systemizing* and higher interest in *empathizing* as fundamental differences that make efforts toward equal representation in tech “unfair, divisive, and bad for business.” Damore was fired for his statements, but the debate itself was harmful. One woman engineer voiced, “I’m exhausted by having this same damn argument over and over again [. . .] and the amount of time and energy I keep having to spend to counter it” (Cowansage, 2017). Biologically essentialized accounts of gender differences work not only to *explain*, but also to *sustain*, gender differences by threatening women’s sense of fit, belonging, and ability to be successful in male-dominant fields (Bian et al., 2018; Cheryan & Markus, 2020; Dar-Nimrod & Heine, 2006; Fine, 2012).

Damore’s memo illustrates the far-reaching consequences of empathizing-systemizing (E-S) theory (Baron-Cohen, 2002, 2004) and its claims of innate sex differences. Given the potential cost of these claims, a close look at the evidence supporting E-S theory is warranted.

In the present work, we examine (a) the degree to which measures developed to support E-S theory include activities for which men and women are perceived to have different opportunities to learn, and (b) whether perceived learning affordances, more than perceived genetic advantages, better predict gender differences on these activities.^1^

## Systemizing and Empathizing: Biological Advantages or Sociocultural Affordances?

E-S theory, initially developed as a theory of autism (Baron-Cohen, 2002), posits that autism spectrum conditions entail higher *systemizing*, or “the drive to analyze the variables in a system, to derive the underlying rules that govern the behaviour of a system [. . .] and the drive to construct systems” (Baron-Cohen et al., 2003, p. 361) and lower *empathizing*, or “the ability to tune into how someone else is feeling, or what they might be thinking [. . .] understand the intentions of others, predict their behavior, and experience an emotion triggered by their emotion” (Baron-Cohen & Wheelwright, 2004, p. 163). In extending E-S theory beyond autism, Baron-Cohen defines the *male brain* as having significantly more systemizing than empathizing ability and the *female brain* as having significantly more empathizing than systemizing ability (Baron-Cohen, 2002, 2004, 2009). Thus, Baron-Cohen (2002, 2004, 2009) asserts that autism represents an exaggerated version of the typical male profile, but the clear assumption of *female/male brain* terminology is that differences in systemizing and empathizing are rooted in biological differences by sex, more than sociocultural differences by gender.

Sociocultural accounts of gender differences, in contrast, assume that gender is performed and constructed through shared social scripts cued by the environment and reinforced by perceiver expectations, cultural stereotypes, and targets’ own self-schemata (Deaux & Major, 1987). Those scripts and stereotypes might have first developed historically in light of physical differences between men and women (e.g., in size and strength, reproductive characteristics) that constrained gender roles (Eagly & Wood, 2012). But once in place, these stereotypes provide persistent prescriptive and proscriptive norms for what is considered appropriate behavior for women and men (Brescoll, 2016; Rudman & Glick, 2001; Vandello & Bosson, 2013). Through the repeated performance of gendered behavior and roles, individuals reinforce the notion of *men* and *women* as binary gender categories with unique psychological drives (Butler, 1990; Morgenroth & Ryan, 2018). From this perspective, women develop empathizing and men develop systemizing skills, in part, to the degree that others expect them to have an inherent interest in these different skills (Aday, 2023).

Although the true origins of gender differences likely involve a complex interplay of biological and sociocultural factors, lay people and scientists often preference one account or the other. As seen in Damore’s memo, essentialized biological interpretations of gender differences can be harmful. The goal of the present article is not to directly identify the origins of gender differences in systemizing and empathizing tendencies but rather to examine whether self-report scales used to document these tendencies reflect gender differences in perceived learning affordances more than perceived genetic advantages.

## Evidence of Gender Differences in Systemizing and Empathizing

What is the evidence for gender differences in systemizing and empathizing? In contrast to Baron-Cohen’s assertions of male and female brains, evidence does not reveal clear sexual dimorphism in the brain (Joel et al., 2015) or hormones (Hyde et al., 2019). Furthermore, meta-syntheses of psychological data suggest that men and women are much more similar than different (Hyde, 2014; Zell et al., 2015). That said, one of the largest effects is a self-reported preference for people versus things, a distinction similar to empathizing/systemizing (Fine, 2010, 2012; Gillis-Buck & Richardson, 2014; Grossi & Fine, 2012). Specific to E-S measures, men score higher than women on the Systemizing Quotient (SQ) in both its original (*d =* .59, *N =* 278; Baron-Cohen et al., 2003) and shortened form (*d =* .95; *N =* 723, Wakabayashi et al., 2006). Likewise, women score higher than men on the Empathizing Quotient (EQ) in both its original (*d = −*.50, *N =* 197; Baron-Cohen & Wheelwright, 2004) and shortened form (*d = −*.63; *N =* 1,038, Wakabayashi et al., 2006).

In contrast to these self-report differences, behavioral evidence is much weaker. Consider the Embedded Figures Task (EFT; Witkin et al., 1971) and the Reading the Mind in the Eyes Test (RMET; Baron-Cohen et al., 2001)—two key measures identified by Baron-Cohen (2002, 2009) as behavioral indicators of systemizing and empathizing, respectively (Chapman et al., 2006). Cross-cultural studies find no gender difference in EFT (systemizing) performance (Kühnen et al., 2001); the RMET (empathizing) also yields small effects in meta-analyses (*g* = 0.18; Kirkland et al., 2013) and large-scale studies (*g* = −0.10; Schroeter et al., 2022). Given that evidence of differences is primarily tied to self-report measures, our interest is in whether those measures are perceived to be capturing gender differences in genetic advantages, as E-S theory asserts.

## SQ and EQ Scales

The SQ (e.g., “When I look at a building, I am curious about the precise way it was constructed”; Baron-Cohen et al., 2003) and EQ (e.g., “I find it easy to put myself in somebody else’s shoes”; Baron-Cohen & Wheelwright, 2004) were developed to measure variability on these dimensions. In each measure, participants rate their agreement with 40 statements and 20 filler items. Wakabayashi et al. (2006) developed short scales of each construct (the 25-item SQ-Short and 22-item EQ-Short) based on factor analyses of the SQ and EQ measures. Although other forms of the SQ and EQ have been developed (e.g., revised EQ, Muncer & Ling, 2006; the Children’s SQ and EQ, Auyeung et al., 2009), we focus on self-report measures developed by Baron-Cohen for adult samples.

Gender differences found on the SQ and EQ are often interpreted as supporting E-S theory’s claims of inherent sex differences. For example, in Archer’s (2019, p. 35) review entitled, “the reality and evolutionary significance of human sex differences,” the first piece of evidence provided under the heading of “Evidence for evolutionary origins” cites evidence from the SQ: “In a study of 53 nations, men consistently scored much higher than women on systemizing (Manning et al., 2010).” In contrast to this evolutionary interpretation, such effects could reflect differences in opportunities afforded to men and women to learn the skills referenced in the items (e.g., SQ-Short Item 2: “If there was a problem with the electrical wiring in my home, I’d be able to fix it myself”) more than genetic advantages that men and women have for systemizing and empathizing activities.

Indeed, prior research documents gender gaps in learning opportunities afforded to boys and girls during childhood (Lytton & Romney, 1991; Tenenbaum & Leaper, 2003). One study conducted among parents of elementary school children found parents were more likely to encourage daughters to learn cooking and homemaking skills and to encourage sons to work on or play with a computer outside of school (as described in Eccles et al., 2000). Such research provides a different interpretation for observed gender differences on SQ and EQ.

## Prior Efforts to Reduce Gender Bias on the SQ and EQ

Concurrent to Wakabayashi et al.’s (2006) development of the SQ-Short, Wheelwright et al. (2006) used the same dataset to create the SQ-R by adding systemizing activities from gender-neutral or feminine domains (e.g., grammar, animals, and family). This revised scale yielded a reduced but still moderate, gender difference (*d =* .49, *N =* 1,761; Wheelwright et al., 2006). In addition, Allison et al. (2015) found that 41% of SQ-R items functioned differently across gender. After eliminating these items, this debiased SQ measure yielded a reduced but still moderate gender difference (*d =* .53, *N =* 4,058). In other work, no EQ items functioned differently for men and women (Allison et al., 2011)

Although prior efforts to debias systemizing measures present a promising step, there are three key reasons for continued examination of these scales. First, although published nearly a decade ago, Allison et al.’s (2015) debiased version of the SQ scale is not widely cited and the addition of gender-neutral activities on the SQ-R does not directly address concerns that original items might be partly assessing variance in men’s and women’s different learning affordances. Second, assessment experts have critiqued the psychometric properties of EQ and have cautioned against using the EQ scale without further empirical validation (Harrison et al., 2022). Third, despite prior critiques of these measures, researchers continue to cite differences in the SQ and the EQ to support conclusions about innate sex differences (Archer, 2019), although self-reported measures can never reveal the etiology of observed differences in the constructs they assess. Given these concerns, we tested the degree to which SQ and EQ might measure gender differences that people perceive as reflective of learning affordances, rather than innate differences.

## Overview of Current Research

We hypothesized that observed gender differences in systemizing and empathizing would be better predicted by people’s perceptions of men’s and women’s learning affordances for activities referenced in the SQ and EQ, more than their perceptions of innate gender differences on the same activities. We tested this with the SQ- and EQ-Short, the two scales that have received some psychometric validation using factor analysis and are widely used given their shorter format. We addressed two questions:

**Research Question 1:** Do the SQ- and EQ-Short ask about activities perceived to reflect men’s and women’s innate differences and/or different learning affordances?
**Research Question 1:** Do perceived innate differences or learning affordances better predict the size of the gender difference observed on the SQ- and EQ-Short items?

To measure perceptions of learning affordances and genetic differences on SQ and EQ items, we adopted a “wisdom of crowds” approach to assess how diverse and expert samples perceive the activities assessed in these scales (Larrick et al., 2011). In a similar way, Swim (1994) demonstrated that people accurately estimate gender differences measured through meta-analyses. We first conducted a target study to obtain an estimate of the gender differences on each SQ- and EQ-Short item among representative samples from the United States and United Kingdom (Study 1). We next conducted two coding studies to examine how lay coders (Study 2) and experts in human behavior (i.e., psychology journal editorial board members, Study 3) estimate the learning affordances and genetic advantages for each of the activities referenced in the items. We tested the hypothesis that those items with the largest gender differences in the target sample (Study 1) would be judged by lay perceivers and experts (in Studies 2 and 3) as having larger gender affordances, more than genetic advantages.

## Study 1: Estimating SQ and EQ Gender Differences

Study 1 estimated the size of the gender difference on each item of the SQ- and EQ-Short scales (Wakabayashi et al., 2006) among two nationally representative samples of participants from the United Kingdom (where the scales were originally developed) and the United States (where coders for Studies 2 and 3 were largely based). As there were no significant country differences on item-level analyses after Bonferroni correction, we assigned each item a score based on the effect size (Cohen’s *d* for the gender difference) observed in the combined sample. This item-level score was then used as the outcome measure in Studies 2 and 3. Given the descriptive nature of Study 1, we did not preregister hypotheses.

### Method

All data, materials, and analysis codes for Studies 1 to 3 are available at osf.io/pyfk4/?view_only=941f5eb11e5642e0b1bd8b6f3ec47cbe.

#### Participants

Our final sample included 624 adults (302 men, 315 women) recruited through Prolific (*N*_US_ = 313, *N*_US_ = 306). We utilized Prolific’s nationally representative sampling option, which employs a stratified sampling technique to match the demographic composition of the country on gender, age, and ethnicity (see SOM for sample comparisons to census data). Descriptive information by country is provided in Table 1. Additional participants were excluded from the final sample for failing an instructional attention check (*n* = 37; Oppenheimer et al., 2009; see SOM). A sensitivity analysis indicated we were able to detect effects above *d =* .23 with 80% power (see SOM for details).

**Table 1.** Sample Demographics by Region (Study 1).

Demographi Characteristic	United States *N* = 313 *N* (%)	United Kingdom *N* = 306 *N* (%)	Combined sample (%)
Gender			
Man	152 (48.56)	150 (49.02)	302 (48.40)
Woman	159 (50.80)	156 (50.98)	315 (50.48)
Nonbinary	2 (0.64)	0 (0)	2 (0.32)
Racial/ethnic background			
Black or African	43 (13.74)	16 (5.23)	59 (9.46)
East Asian	12 (3.83)	6 (1.96)	18 (2.88)
Hispanic or Latino/a	12 (3.83)	1 (0.33)	13 (2.08)
Indigenous	3 (0.96)	0 (0)	3 (0.48)
Middle Eastern or Arabic	1 (0.32)	2 (0.65)	3 (0.48)
South Asian	6 (1.92)	22 (7.19)	28 (4.49)
Southeast Asian	7 (2.24)	1 (0.33)	8 (1.28)
White^a^	208 (66.45)	237 (77.45)	445 (71.31)
Not Listed	3 (0.96)	19 (6.21)	22 (3.53)
Multiracial	18 (5.75)	2 (0.65)	20 (3.21)
Age	3.24 (1.65)	3.53 (1.31)	3.38 (1.50)

a In the original measures for Studies 1 to3, the option “White” was listed as “White or Caucasian.”

### Procedure and Measures

After passing the initial attention check and providing consent, participants completed the SQ-Short (25 items) and EQ-Short (22 items; see Table 2), presented in random order. They rated their agreement with each statement on a scale ranging from 1 (Strongly disagree) to 7 (Strongly agree).^2^ Finally, participants provided basic demographic information. Participants received £2.50 (UK) and $2.73 (US) for completing the survey.

**Table 2.** Items, Descriptive Statistics by Gender, and Effect Size for Gender Difference (Study 1).

Variable	Item text	Women	Men	Gender difference
		*M* (*SD*)	*M* (*SD*)	*d* [95% CI[
Systemizing (Composite)		4.07 (0.87)	4.85 (0.82)	0.92 [.75, 1.09]
Sys2	If there was a problem with the electrical wiring in my home, I’d be able to fix it myself.	2.02 (1.40)	3.79 (1.99)	1.04 [.87, 1.21]
Sys5	I am fascinated by how machines work.	4.00 (1.63)	5.35 (1.37)	0.89 [.72, 1.05]
Sys22	I can easily visualize how the motorways in my region link up.	3.52 (1.69)	4.72 (1.72)	0.71 [.55, .87]
Sys14	I find it easy to grasp exactly how odds work in betting.	3.32 (1.65)	4.48 (1.69)	0.70 [.54, .86]
Sys3	I rarely read articles or web pages about new technology. (R)	4.15 (1.71)	5.24 (1.65)	0.65 [.49, .81]
Sys25	I am not interested in understanding how wireless communication works. (R)	3.87 (1.78)	4.89 (1.62)	0.60 [.44, .76]
Sys8	If I were buying a computer, I would want to know exact details about its hard disk drive capacity and processor speed.	4.94 (1.87)	5.91 (1.45)	0.58 [.42, .74]
Sys13	If I were buying a stereo, I would want to know about its precise technical features.	4.20 (1.83)	5.20 (1.71)	0.56 [.40, .72]
Sys9	I find it difficult to read and understand maps. (R)	4.94 (1.69)	5.80 (1.48)	0.54 [.38, .70]
Sys4	I do not enjoy games that involve a high degree of strategy. (R)	4.38 (1.66)	5.24 (1.57)	0.53 [.37, .69]
Sys6	In math, I am intrigued by the rules and patterns governing numbers.	3.52 (1.87)	4.49 (1.80)	0.53 [.37, .69]
Sys1	If I were buying a car, I would want to obtain specific information about its engine capacity.	4.51 (1.74)	5.36 (1.55)	0.52 [.36, .68]
Sys23	When I’m in a plane, I do not think about the aerodynamics. (R)	3.26 (1.68)	4.10 (1.82)	0.48 [.32, .64]
Sys7	I find it difficult to understand instruction manuals for putting appliances together. (R)	4.90 (1.63)	5.59 (1.39)	0.45 [.29, .61]
Sys18	When traveling by train, I often wonder exactly how the rail networks are coordinated.	3.30 (1.72)	3.99 (1.68)	0.41 [.25, .57]
Sys16	When I look at a building, I am curious about the precise way it was constructed.	3.51 (1.70)	4.20 (1.73)	0.40 [.24, .56]
Sys11	I find it difficult to learn my way around a new city. (R)	4.28 (1.64)	4.88 (1.62)	0.37 [.21, .53]
Sys17	I find it difficult to understand information the bank sends me on different investment and saving systems. (R)	4.73 (1.65)	5.29 (1.53)	0.35 [.19, .51]
Sys21	When I look at a mountain, I think about how precisely it was formed.	3.23 (1.65)	3.70 (1.66)	0.28 [.12, .44]
Sys12	I do not tend to watch science documentaries on television or read articles about science and nature. (R)	4.83 (1.84)	5.26 (1.68)	0.25 [.09, .41]
Sys24	I am interested in knowing the path a river takes from its source to the sea.	3.95 (1.76)	4.36 (1.72)	0.24 [.08, .40]
Sys20	When I hear about the weather forecast, I am not very interested in the meteorological patterns. (R)	4.03 (1.70)	4.38 (1.71)	0.21 [.05, .37]
Sys10	When I look at a piece of furniture, I do not notice the details of how it was constructed. (R)	4.26 (1.75)	4.61 (1.57)	0.21 [.05, .37]
Sys19	If I were buying a camera, I would not look carefully into the quality of the lens. (R)	5.14 (1.66)	5.42 (1.68)	0.16 [.00, .32]
Sys15	I am not very meticulous when I carry out D.I.Y. (R)	4.86 (1.63)	4.90 (1.65)	0.02 [−.14, .18]
Empathizing (composite)		5.16 (0.70)	4.71 (0.87)	−0.57 [−.73, −.41]
Emp22	I tend to get emotionally involved with a friend’s problems.	4.99 (1.33)	4.09 (1.57)	−0.62 [−.78, −.46]
Emp17	Other people often say that I am insensitive, though I don’t always see why. (R)	5.77 (1.30)	4.93 (1.55)	−0.59 [−.75, −.43]
Emp13	Other people tell me I am good at understanding how they are feeling and what they are thinking.	5.26 (1.26)	4.49 (1.49)	−0.56 [−.72, −.40]
Emp15	Friends usually talk to me about their problems as they say that I am very understanding.	5.48 (1.24)	4.73 (1.50)	−0.54 [−.70, −.38]
Emp7	It is hard for me to see why some things upset people so much. (R)	5.21 (1.54)	4.40 (1.63)	−0.51 [−.67, −.35]
Emp18	I can tune into how someone else feels rapidly and intuitively.	5.24 (1.17)	4.61 (1.40)	−0.49 [−.65, −.33]
Emp2	I really enjoy caring for other people.	5.36 (1.32)	4.78 (1.56)	−0.40 [−.56, −.24]
Emp10	I am quick to spot when someone in a group is feeling awkward or uncomfortable.	5.57 (1.08)	5.09 (1.32)	−0.40 [−.56, −.24]
Emp16	I can sense if I am intruding, even if the other person doesn’t tell me.	5.58 (1.09)	5.20 (1.23)	−0.33 [−.49, −.17]
Emp9	I am good at predicting how someone will feel.	4.99 (1.20)	4.59 (1.30)	−0.32 [−.48, −.16]
Emp8	I find it easy to put myself in somebody else’s shoes.	5.23 (1.35)	4.79 (1.44)	−0.31 [−.47, −.15]
Emp11	I can’t always see why someone should have felt offended by a remark. (R)	4.68 (1.66)	4.20 (1.46)	−0.31 [−.47, −.15]
Emp5	In a conversation, I tend to focus on my own thoughts rather than what my listener might be thinking. (R)	4.53 (1.42)	4.15 (1.40)	−0.27 [−.43, −.11]
Emp1	I can easily tell if someone else wants to enter a conversation.	5.50 (1.04)	5.23 (1.24)	−0.24 [−.40, −.08]
Emp14	I can easily tell if someone else is interested or bored with what I am saying.	5.54 (1.06)	5.27 (1.24)	−0.23 [−.39, −.07]
Emp3	I find it hard to know what to do in a social situation. (R)	4.75 (1.56)	4.45 (1.67)	−0.18 [−.34, −.02]
Emp20	I can tell if someone is masking their true emotion.	4.95 (1.17)	4.72 (1.29)	−0.18 [−.34, −.02]
Emp4	I often find it difficult to judge if something is rude or polite. (R)	5.58 (1.34)	5.35 (1.31)	−0.17 [−.33, −.01]
Emp12	I don’t tend to find social situations confusing.	4.74 (1.47)	4.49 (1.64)	−0.16 [−.32, .00]
Emp19	I can easily work out what another person might want to talk about.	4.75 (1.22)	4.54 (1.33)	−0.16 [−.32, .00]
Emp6	I can pick up quickly if [sic] someone says one thing but means another.	5.04 (1.21)	4.86 (1.23)	−0.15 [−.31, .01]
Emp21	I am good at predicting what someone will do.	4.72 (1.20)	4.54 (1.24)	−0.15 [−.31, .01]

*Note.* Items marked with (R) are reverse-scored. Negative *d* scores indicate women scored higher; positive *d* scores indicate men scored higher. CI = confidence interval.

## Results

### Gender Difference in Systemizing and Empathizing Composites

**Figure 1:**
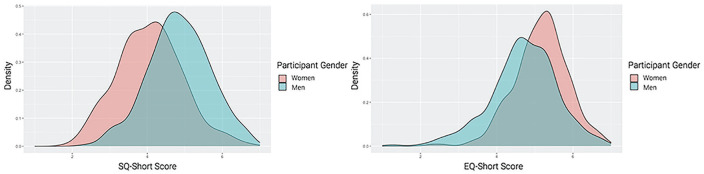
Distribution of Scores on the SQ-Short and EQ-Short (Study 1) *Note.* SQ = Systemizing Quotient; EQ = Empathizing Quotient.

First, to estimate gender differences in systemizing and empathizing, we calculated an average score for each person based on their ratings of all systemizing items and all empathizing items. One participant was missing data on systemizing items and did not receive a composite score. Both composites showed good reliability, *α*_SQ_ = .90, *α*_EQ_ =.92, and were uncorrelated (*r =* .02, *p =* .702). As shown in Table 2, men scored significantly higher than women on the systemizing quotient, whereas women scored significantly higher than men on the empathizing quotient. Figure 1 shows the distribution of scores on the SQ-Short and EQ-Short by participant gender.

### Gender Difference in Systemizing and Empathizing Items

Next, we obtained an effect size estimate for the gender difference on each item (*N =* 617; see Table 2).^3^ We used an arbitrary cut-off of *d =* .30 (reflecting a small but meaningful effect size) to bin items into three categories: women scored higher than men (*d* < −.30), men and women scored relatively equal (−.30 < *d* < .30), and men scored higher than women (*d* > .30).^4^ Using these cut-offs, men scored higher than women on the majority (70%) of systemizing items and women scored higher than men on just over half (55%) of empathizing items. Within each scale, the size of the gender differences varied considerably. For example, on the SQ-Short, the item with the smallest effect size was Sys15: “I am not very meticulous when I carry out D.I.Y.” (reverse-scored, *d =* .02) and the item with the largest effect size was Sys2: “If there was a problem with the electrical wiring in my home, I’d be able to fix it myself,” *d =* 1.04). On the EQ-Short, the item with the smallest effect size was Emp21: “I am good at predicting what someone will do,” *d = −*.15) and the item with the largest effect size was Emp22: “I tend to get emotionally involved with a friend’s problems,” *d = −*.62). In addition, the average size of the gender differences on individual items (SQ-Short: M*_d_ =* .47, *SD* = .23; EQ-Short: M*_d_ = −*.33, *SD* = .16) tended to be smaller than the size of the gender difference on the composites (SQ-Short: *d =* .92; EQ-Short: *d = −*.57; see Eagly & Revelle, 2022).

## Discussion

Study 1 revealed that, as expected, men scored significantly higher than women on systemizing and women scored significantly higher than men on empathizing. However, the size of these gender differences varied considerably across items within the scale, possibly reflecting differences in specific activities described in the items. Indeed, if some items capture gender differences that are more due to learning affordances, than to genetic differences, then perceived affordances to learn these skills should predict the size of the gender difference observed, more than perceptions of genetic advantages. As reviewed above, we used a wisdom of crowds approach (Larrick et al., 2011) to test hypotheses about perceived gender differences among a diverse sample of lay coders (Study 2) and experts in psychology (Study 3), as having diverse perspectives and expertise are the two conditions under which crowds are assumed to be wise.

## Study 2

The goal of Study 2 was to assess the degree to which gender differences observed on SQ- and EQ-Short items in Study 1 are related to perceived patterns of gender-based learning affordances in the activities assessed by the SQ- and EQ-Short. We asked a diverse sample of lay coders to rate activities from the SQ- and EQ-Short on (a) the estimated gender difference, (b) whether that activity is likely to be innate versus learned, (c) whether men or women have more affordances to learn that activity, and (d) how reflective of genetic sex differences each activity is. Since this initial study was exploratory, we did not preregister our hypotheses nor analytic strategy.

### Method

#### Participants

Our final sample included *N =* 199 coders from the United States and Canada recruited through Amazon’s Mechanical Turk. Table 3 provides a summary of participant demographics. Participants were excluded from this final sample for failing the same attention check used in Study 1 (*n* = 23). Although a minimum of two coders is recommended for intercoder reliability (O’Connor & Joffe, 2020), given the subjective nature of our ratings, we aimed to collect a large and diverse sample of coders (about 50 coders per activity). In our final sample, each rating had between *N =* 49 and 77 (*M =* 62.85, *SD =* 9.94) participant coders. Ratings showed good interrater reliability, intraclass correlation = .90, 95% confidence interval (CI): [.88, .92] (one-way random effects computed with absolute agreement and multiple raters/measurements; Koo & Li, 2016; Shrout & Fleiss, 1979).

**Table 3:** Sample Demographics (Study 2.)

Demographic Characteristic	*N* (%)
Gender	
Man	99 (49.75)
Woman	98 (49.25)
Racial/ethnic background	
Black or African	33 (16.58)
East Asian	6 (3.02)
Hispanic or Latino/a	11 (5.53)
Indigenous	1 (0.50)
South Asian	6 (3.02)
Southeast Asian	2 (1.01)
White	132 (66.33)
Not Listed	1 (0.50)
Multiracial	5 (2.51)
	*M* (*SD*)
SES (1 = Lowest SES, 10 = Highest SES)	6.04 (2.13)
Age	33.89 (10.13)

*Note.* SES = socioeconomic status.

### Procedure and Measures

Participants received $1.50 to participate in a study titled “Activity, Interest, and Ability Ratings.” After providing consent, participants rated a subset of 15 (of 47 total) activities abstracted from the SQ and EQ-Short items (e.g., “getting emotionally involved with a friend’s problems” (see SOM for materials). Of the 15 activities shown to each coder, five were activities that women scored higher on, five activities were gender neutral, and five were activities that men scored higher on (estimates using cut-offs described in Study 1). Participants rated each activity on:

1. Estimated gender differences: “To what degree do men and women differ on [activity]?” ranging from 1 (WOMEN are higher) to 4 (It is equal) to 7 (MEN are higher).
2. Learned vs. innate attributions: “To what degree is [activity] reflective of a preference/skill one is born with vs. a preference/skill one learns through experience?” ranging from 1 (More of a skill one is BORN WITH) to 4 (It is equal) to 7 (More of a skill one LEARNS through experience).
3. Gendered learning affordances: “Who has more opportunities to learn about [activity]?” ranging from 1 (WOMEN have more opportunities to learn this) to 4 (It is equal) to 7 (MEN have more opportunities to learn this).
4. Assumed genetic sex differences: “To what degree is [activity] reflective of innate, genetic differences between men and women?” ranging from 1 (Not at all) to 4 (Somewhat) to 7 (Very much).

The order of the last two ratings was counterbalanced between coders. The full list of measures collected in Study 2 is available in the SOM.^5^

## Results

**Figure 2.**
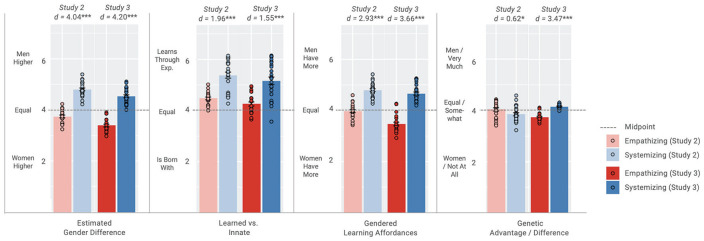
Lay Coder (Study 2) and Expert Ratings (Study 3) for Scale Items and Comparison Against Midpoint. *Note.* Effect sizes (*d*) reported for the difference between systemizing and empathizing items. *p < .05. **p < .01. ***p < .001.

For item-level analyses, we assigned each item a value based on the unweighted mean of coder ratings for that item^6^ (Lorenz et al., 2011). This yielded 47 datapoints (25 SQ-Short items, 22 EQ-Short items) per rating dimension, each with a value reflecting the average rating for a given activity on that dimension. Table 4 and Figure 2 provide a summary of coder ratings. Mean ratings on each activity and results for the corresponding test against the scale midpoint are provided in the SOM. Coder gender sometimes affected item ratings but not how these ratings predicted gender differences in the target sample (see SOM).

**Table 4.** Descriptive Statistics, Effect Size for Difference From Midpoint, and N items Significantly Below or Above Midpoint (Study 2).

Rating dimension	SQ-short activities				EQ-short activities			
	*M* (*SD*)	Difference from midpoint (*d*) [95% CI]	Items below midpoint *N* (%)	Items above midpoint *N* (%)	*M* (*SD*)	Difference from midpoint (*d*) [95% CI]	Items below midpoint *N* (%)	Items above midpoint *N* (%)
Estimated gender difference	4.80 (0.27)	2.97*** [2.56, 3.37]	0 (0)	24 (96)	3.74 (0.25)	1.02*** [.72, 1.31]	6 (27.27)	2 (9.09)
Learned vs. innate attributions	5.38 (0.59)	2.33*** [1.97, 2.69]	0 (0)	24 (96)	4.47 (0.25)	1.86*** [1.53, 2.19]	0 (0)	15 (68.18)
Gendered learning affordances	4.80 (0.28)	2.86*** [2.46, 3.25]	0 (0)	24 (96)	3.98 (0.28)	0.08 [−0.20, 0.36]	2 (9.90)	4 (18.18)
Genetic differences	3.85 (0.31)	N/A	N/A	N/A	4.04 (0.30)	N/A	N/A	N/A

*Note.* SQ = Systemizing Quotient; EQ = Empathizing Quotient; Below Midpoint = Women Higher, More Innate; Above Midpoint = Men Higher, More Learned; CI = confidence interval.

*** *p* < .001.

### Estimated Gender Difference

Coders rated men and women as differing significantly on systemizing and empathizing activities, *t*(44.78)^7^ = −13.87, *p* < .001, *d =* 4.04, with men rated as higher on systemizing activities and women rated as higher on empathizing activities (as revealed by one sample *t*-tests comparing means to the scale midpoint, see Table 4). At the item level, coders rated men significantly higher on all but one systemizing activity and rated women significantly higher on 27.27% of empathizing activities. Men were also rated significantly higher on two empathizing activities prior to reverse-scoring (“Being insensitive,” “Focusing on one’s own thoughts rather than what their listener might be thinking.”).

### Learned Versus Innate Attributions

Coders rated systemizing activities as being more learned through experience than empathizing activities, *t*(33.37) = −7.00, *p* < .001, *d =* 1.96. Both systemizing and empathizing activities were rated as being relatively more learned through experience than a skill one is born with (Table 4, tests against scale midpoint). At the item level, coders rated all but one systemizing activity and most (68.18%) empathizing activities as significantly more learned through experience than a skill one is born with. No activities were rated as being significantly more innate than learned through experience.

### Gendered Learning Affordances

Coders rated men and women as differing significantly in their affordances to learn systemizing and empathizing activities, *t*(44.08) = −10.01, *p* < .001, *d =* 2.93. Coders rated men as having significantly more affordances than women to learn systemizing activities, whereas they assumed men and women have equal affordances to learn empathizing activities (Table 4, tests against scale midpoint). At the item level, coders rated all but one systemizing activity as reflecting more affordances to men. Unexpectedly, coders also rated 18.18% of empathizing activities as providing more affordances to men, and only two empathizing activities were rated as providing more affordances to women.

### Genetic Differences

Finally, both systemizing and empathizing activities were rated as being somewhat reflective of innate genetic differences (Table 4), but less so for systemizing than empathizing, *t*(44.48) = 2.13, *p* = .039, *d =* 0.62. Since this item was rated from not at all to very much, comparisons to the midpoint were ambiguous, a limitation we rectified in Study 3.

### Item-Level Analyses Predicting Observed Gender Differences From Coder Ratings

Having established that systemizing items, in particular, were perceived to capture learnable skills that men have more opportunities to learn, we next tested whether the item-level perceptions measured by this independent sample of lay coders (Study 2) predicted the size of the gender differences across items measured in Study 1.

### Analytic Strategy

As in the results above, each item on the SQ- and EQ-Short was assigned a value (for each of the four measures) based on aggregated coder ratings in this sample. We also assigned each item a value based on the effect size for the gender difference observed on that item from Study 1 (positive values = men higher, negative values = women higher). Each model tested the main effect of coder rating dimension (continuous, standardized) and subscale (categorical: systemizing vs. empathizing), as well as their interaction, as predictors of the observed gender difference in Study 1. To derive the main effects of coder ratings on observed gender differences across subscale, we contrast-coded our subscale variable (systemizing = 0.5, empathizing = −0.5), and dummy-coded categorical predictors to examine simple slopes.

### Estimated Gender Difference

To validate the wisdom of the crowds approach, we first tested whether participant coders (Study 2) accurately estimated the true gender difference in each activity (Study 1). As expected (and consistent with prior work by Swim, 1994), coder ratings of gender differences positively predicted the observed gender differences, *β* = .64, *p* < .001, 95% CI [.40, .88], such that coders accurately tracked the size of the gender difference on each activity. There was no interaction by subscale, *β* = .20, *p* = .411, 95% CI [−.28, .68].

### Learned vs. Innate Attributions

Next, we examined how coders’ ratings of learned versus innate attributions for each activity (Study 2) predicted the observed gender difference on the items (Study 1). There was a main effect of coder ratings on the observed gender difference, *β* = 0.31, *p* = .022, 95% CI [.05, .57], but no significant interaction by subscale (systemizing vs. empathizing), *β* = −.42, *p* = .115, 95% CI [−.95, .11].^8^ Activities that coders judged to be less learned through experience were those on which women scored higher. Recall that all items were coded as being equally or more learned than innate.

### Gendered Learning Affordances

**Figure 3.**
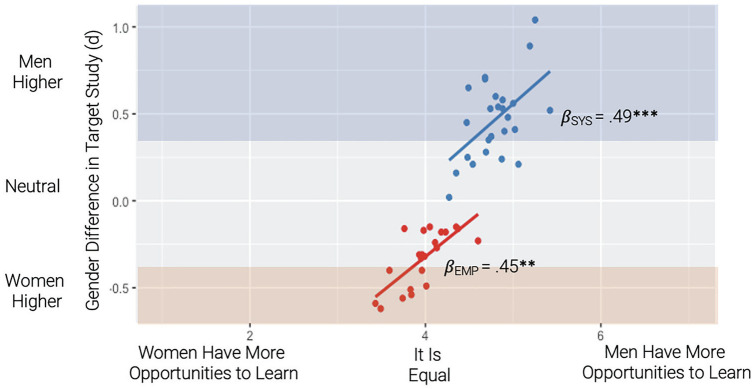
Relationship Between Coder Ratings of Gendered Learning Affordances (Study 2) and Observed Gender Difference (Study 1). *Note.* Beta coefficients represent simple slopes for systemizing and empathizing separately. Axis bands represent a gender difference with (*d* > .30; blue) or women scoring higher (*d* < -.30; red) in Study 1.

We next examined the relationship between coders’ perceptions of men's and women’s relative affordances to learn systemizing and empathizing activities (Study 2) and the observed gender difference on corresponding items (Study 1). There was a significant main effect of affordances, *β* = .47, *p* < .001, 95% CI [.27, .67]. There was no moderation by subscale (systemizing vs. empathizing), *β* = .04, *p* = .830, 95% CI [-.35, .44]. Figure 3 reveals that systemizing activities that men have more (perceived) affordances to learn are those that men scored higher on (*β* = .49, *p* < .001), and empathizing activities that women have more (perceived) affordances to learn are those on which women scored higher (*β* = .45, *p* < .05). Axis bands represent a gender difference with [*d* > .30; blue] or women scoring higher [*d* < −.30; red] in Study 1).

### Genetic Differences

We next examined the relationship between coders’ ratings of genetically based sex differences (Study 2) and observed gender differences on the items (Study 1). There was no significant main effect of coder ratings on the observed gender difference, *β* = −.02, *p* = .804, 95% CI [−.15, .12], and no significant interaction by subscale (systemizing vs. empathizing), *β* = .27, *p* = .054, 95% CI [.00, .55].

## Discussion

Study 2 provides initial evidence that the gender differences observed on the SQ and EQ-Short scales could be partly assessing men’s and women’s different affordances to learn the activities referenced in the items. First, all activities included in the SQ and EQ-Short measures are rated by lay coders to be equally or more learnable than innate. Coders also believed that men had significantly greater affordances to learn systemizing activities and believed men and women have equal affordances to learn empathizing activities. Coders were able to predict the size of the gender difference in each activity with relative accuracy, providing some validity for wisdom of crowds approach to estimating true gender differences in the population.

Across both subscales, there was an intuitive correspondence between the gendered affordances of these activities (as rated by coders in this study) and the observed gender differences assessed in Study 1. Coders rated all systemizing activities as being those where men have greater affordances to learn, and those activities rated as having stronger affordances for men were those where men scored higher than women (Study 1). Although empathizing items were not rated as generally providing more affordances to women, those items where women were seen as having greater affordances were the items where women scored higher than men (Study 1). In contrast, coder attributions of sex differences to genetic advantages did not significantly predict the magnitude of gender difference observed on the items.

Although these findings provide some evidence that the SQ-Short and (to a lesser extent) EQ-Short are, in part, indexing patterns of learning affordances perceived to vary across gender, there were three main limitations of this study. First, although it is informative to track lay perceptions, those with expertise in human psychology might be in a better position to estimate the causal factors underlying gender differences. We reasoned that expert editorial board members at influential psychology journals across a variety of subfields would satisfy both conditions (diversity and expertise) under which crowds can be wise (Larrick et al., 2011).

Second, although we found no evidence that coders’ ratings of genetic influence predicted observed gender differences, our measure lacked parallelism to the learning affordances item. In Study 3, we reworded this item to ask about genetic advantages for men or women to directly test these two ratings as competing predictors of the observed gender difference in Study 1. Finally, based on Study 2 results, we preregistered our analytic plan and hypotheses prior to conducting analyses in Study 3.

## Study 3

The goal of Study 3 was to conduct a preregistered replication of Study 2 among a sample of expert coders recruited from editorial boards of influential psychology journals. Experts completed the same rating activities from Study 2. Our preregistered hypotheses included:

**Primary Hypothesis 1:** Experts are able to accurately estimate gender differences in systemizing and empathizing items.
**Primary Hypothesis 2:** The items that experts believe that men have greater opportunities to learn will be those that men score higher on relative to women (and vice versa).
**Primary Hypothesis 3:** The items that experts believe focus on more learnable activities/behaviors will show larger gender differences.
**Exploratory Hypothesis 4:** The items for which experts believe men have a greater genetic advantage will be those that men score higher on relative to women (and vice versa).

### Method

#### Participants

We emailed study invitations to *N =* 612 experts who at the time were editorial board members of influential journals spanning clinical, developmental, evolutionary, gender, general, neuroscience/cognitive, and social/personality psychology. Details about our recruitment strategy and target sample size is provided in the SOM. Table 5 provides information on contact and response rates by subdiscipline and gender.

**Table 5.** Response Rates by Subdiscipline and Gender (Study 3).

Area	Women		% Res	Men		*%* Res	Grand Total		% Res
	*N* Con	*N* Res		*N* Con	*N* Res		*N* Con	*N* Res	
Clinical	50	6	12.00	53	6	11.32	103	12	11.65
Developmental	50	17	34.00	50	8	16.00	100	25	25.00
Evolutionary	27	7	25.93	26	8	30.77	53	15	28.30
Gender	26	3	11.54	25	4	16.00	51	7	13.73
General	51	4	7.84	50	8	16.00	101	12	11.88
Neuro/cognitive	51	4	7.84	50	11	22.00	101	15	14.85
Social/personality	51	11	21.57	52	19	36.54	103	30	29.13
Grand total	306	52	16.99	306	64	20.92	612	116	18.95

*Note.* Con = Contacted; Res = Responded

The final sample of 116 experts was fairly evenly split by gender (see Table 6 for a summary of participant demographics).^9^ Additional participants were excluded from the final sample for not finishing the survey or indicating on a single item at the end of the survey that we should not use their data (*n* = 35). Participants who expressed interest were sent a report of findings postanalysis.

**Table 6:** Sample Demographics (Study 3).

Demographic Characteristic	*N* (%)
Gender	
Man	64 (55.17)
Woman	52 (44.83)
Racial/ethnic background	
Black or African	4 (3.45)
East Asian	1 (0.86)
Hispanic or Latino/a	2 (1.72)
South Asian	2 (1.72)
White	98 (84.48)
Not Listed	2 (1.72)
Multiracial	5 (4.31)
	*M* (*SD*)
SES (1 = *Lowest SES*, 10 = *Highest SES*)	7.84 (0.99)
Age	52.31 (12.70)
Political Orientation (1 = *Extremely Liberal*, 7 = *Extremely Conservative*)	2.16 (1.00)

*Note.* SES = socioeconomic status.

#### Procedure and Measures

After consenting to the study procedures, similar to Study 2, we showed experts a random subset of 24 (of 47 total) activities abstracted from the SQ- and EQ-Short items. We increased the number of activities presented to experts (from 15 in Study 2 to 24 in Study 3) to maximize data with this difficult-to-recruit sample. Of the 24 activities shown to coders, 8 were activities that women tended to score higher on, 8 were activities that tended to be neutral, and 8 were activities that men tended to score higher on (estimates based on data from Study 1 using the same cut-offs described previously). We asked experts to rate each activity on the same dimensions as in Study 2, with two adjustments made to gendered learning affordances and genetic differences, as described below (see SOM for all Study 3 measures). Similar to Study 2, experts in Study 3 showed excellent interrater reliability, ICC = .97, 95% CI [.96, .97] (one-way random effects with absolute agreement and multiple raters/measurements; Koo & Li, 2016; Shrout & Fleiss, 1979).

##### Gendered Learning Affordances

In Study 3, we adapted this measure to say: “When it comes to [activity]. . . Who has more opportunities to learn?” ranging from 1 (*WOMEN have more*) to 4 (*It is equal*) to 7 (*MEN have more*).

##### Genetic Advantages

In Study 3, experts rated the perceived sex-based genetic advantage of each activity (presented in the same block as gendered learning affordances, order counterbalanced between coders): “When it comes to [activity]. . . Who has a genetic advantage?” ranging from 1 (*WOMEN have more*) to 4 (*It is equal*) to 7 (*MEN have more*).

### Results

The same analytic approach in Study 2 was used in Study 3.^10^ All effects for the estimated gender difference and gendered learning affordances held across coder gender and subdiscipline (see SOM for details).

#### Mean Expert Ratings for SQ- and EQ-Short Activities

We did not preregister hypotheses related to mean ratings across SQ and EQ-Short activities. Table 7 and Figure 2 provide a summary of coder ratings. Mean ratings on each activity and results for the corresponding test against scale midpoint is provided in the SOM.

**Table 7.** Descriptive Statistics, Effect Size for the Difference From Midpoint, and N Items Significantly Below or Above Midpoint for SQ- and EQ-Short Activities (Study 3).

Rating dimension	SQ-short activities				EQ-Short activities			
	*M* (*SD*)	Difference from midpoint (*d*) [95% CI]	Items below midpoint *N* (%)	Items above midpoint *N* (%)	*M* (*SD*)	Difference from midpoint (*d*) [95% CI]	Items below midpoint *N* (%)	Items above midpoint *N* (%)
Estimated gender difference	4.53 (0.29)	1.85*** [1.41, 2.28]	0 (0)	24 (96)	3.38 (0.26)	2.41*** [1.93, 2.89]	18 (81.82)	2 (9.09)
Learned vs. innate attributions	5.16 (0.72)	1.62*** [1.20, 2.04]	1 (4)	20 (80)	4.26 (0.38)	.68** [.30, 1.05]	2 (9.09)	11 (50)
Gendered learning affordances	4.66 (0.31)	2.10*** [1.64, 2.55]	0 (0)	25 (100)	3.47 (0.34)	1.58*** [1.16, 1.99]	20 (90.91)	1 (4.55)
Genetic advantages	4.15 (0.09)	1.71*** [1.28, 2.13]	0 (0)	17 (68)	3.74 (0.15)	1.78*** [1.35, 2.21]	20 (90.91)	0 (0)

*Note.* SQ = Systemizing Quotient; EQ = Empathizing Quotient; Below Midpoint = Women Higher, More Innate; Above Midpoint = Men Higher, More Learned; CI = confidence interval.

** *p* < .01. ***p < .001.

#### Estimated Gender Difference

As in Study 2, experts rated men and women as differing significantly on systemizing and empathizing activities, *t*(45) = −14.46, *p* < .001, *d =* 4.20, with men higher on systemizing activities and women higher on empathizing activities (Table 7, tests against scale midpoint). At the item level, experts rated men significantly higher on all but one systemizing activity and, unlike lay coders, experts rated women significantly higher on the majority (81.82%) of empathizing activities. As in Study 2, experts also rated men as significantly higher on two empathizing activities prior to reverse scoring (“Being insensitive,” “Finding social situations confusing.”).

#### Learned vs. Innate Attributions

Like lay coders, experts rated systemizing activities as being significantly more learned through experience compared with empathizing activities, *t*(37.31) = −5.51, *p* < .001, *d =* 1.55. Also mirroring effects with lay coders, experts rated both systemizing and empathizing activities as being more learned through experience than an innate ability one is born with (Table 7, tests against midpoint). At the item level, experts rated most (80%) systemizing activities and half (50%) of empathizing activities as being significantly more learned through experience than a skill one is born with. Unlike in Study 2, experts rated one systemizing activity (“Being intrigued by the rules and patterns governing numbers in math”) and two empathizing activities (“Finding social situations confusing,” “Being insensitive”; both items reverse-scored) as being significantly more innate than learned through experience.

#### Gendered Learning Affordances

As in Study 2, experts rated men and women as differing significantly in their affordances to learn systemizing and empathizing activities, *t*(43.14) = −12.46, *p* < .001, *d =* 3.66. Experts rated men as having significantly more affordances to learn systemizing activities, and unlike lay coders in Study 2, experts also rated women as having significantly more affordances to learn empathizing activities (Table 7, tests against midpoint). At the item level, experts rated every systemizing activity as providing significantly more affordances to men and all but two empathizing activities as providing more affordances to women. One empathizing activity (“Being insensitive”; reverse-scored) was rated as providing more affordances to men prior to reverse-scoring.

#### Genetic Advantages

Experts rated men and women as differing significantly in their genetic advantage on systemizing and empathizing activities, *t*(33.70) = −11.50, *p* < .001, *d =* 3.47. Experts rated men as having a significantly greater genetic advantage in systemizing activities, and women as having a significantly greater genetic advantage in empathizing activities (Table 7, tests against midpoint). At the item level, experts rated the majority (68%) of systemizing activities as affording a genetic advantage to men and all but two empathizing activities as affording a genetic advantage to women.

#### Predicting Observed Gender Differences from Coder Ratings

Next, as preregistered and replicating Study 2, we examined the relationship between expert ratings and gender differences observed in Study 1.

#### Estimated Gender Difference

As in Study 2 and supporting H1, there was a main effect of expert ratings on observed gender differences, *β* = .71, *p* < .001, 95% CI [.48, .95], such that experts accurately tracked the size of the gender difference on each activity. There was no interaction by subscale (systemizing vs. empathizing), *β* = .19, *p* = .419, 95% CI [−0.28, .66].

#### Learned vs. Innate Attributions

In Study 2, we found no evidence that gender differences would be larger for items that experts believe focus on learned (vs. innate) activities/behaviors. We reasoned, however, that experts might show this effect. However, similar to Study 2 and counter to H2, there was no significant effect of coder ratings on the observed gender difference, *β* = .20, *p* = .057, 95% CI [−.01, .41]^11^; and no significant interaction by subscale (systemizing vs. empathizing), *β* = −.38, *p* = .077, 95% CI [−.79, .04].^12^

#### Gendered Learning Affordances

**Figure 4.**
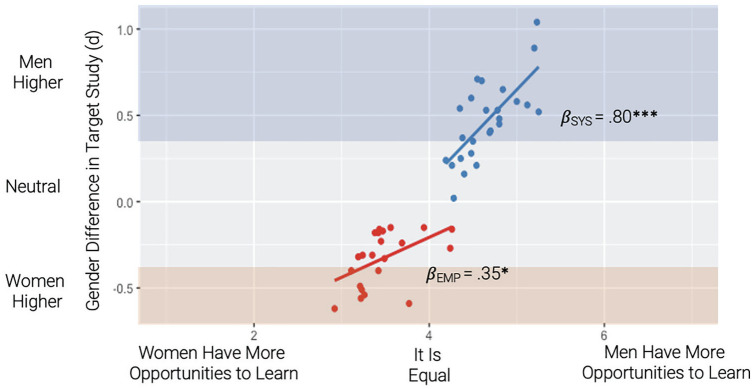
Relationship Between Expert Ratings of Gendered Learning Affordances (Study 3) and Observed Gender Difference (Study 1). *Note.* Beta coefficients represent simple slopes for systemizing and empathizing separately. Axis bands represent a gender difference with (*d* > .30; blue) or women scoring higher (*d* < -.30; red) in Study 1.

As in Study 2 and supporting H3, there was a significant main effect of gendered learning affordances on the observed gender difference in Study 1, *β* = .58, *p* < .001, 95% CI [.36, .80] (Figure 4). The systemizing activities experts rated as providing more affordances to men were also those that men scored higher on; the empathizing activities experts rated as providing more affordances to women were also those that women scored higher on. Unlike in Study 2, there was a significant interaction by subscale (systemizing vs. empathizing) *β* = .45, *p* = .042, 95% CI [.02, .89], revealing that this effect was stronger for systemizing (*β* = .80, *p* < .001, 95% CI [.49, 1.11]) than empathizing (*β* = .35, *p* = .026, 95% CI [.04, .66]).

#### Genetic Advantages

Using the revised measure of perceived genetic advantages in Study 3, we also found support for our exploratory H4. There was a main effect of expert ratings of genetic advantages on the observed gender difference, *β* = .51, *p* < .001, 95% CI [.25, .77], and a significant interaction by subscale, *β* = .60, *p* = .025, 95% CI [.08, 1.12]. Systemizing items rated by experts as providing a genetic advantage to men were also those that men scored higher on (*β* = .81, *p* < .001, 95% CI [.37, 1.24]), but this relationship was not significant for empathizing items (*β* = .21, *p* = .146, 95% CI [−.07, .49]).

#### Testing Competing Predictors: Learning Affordances Versus Genetic Difference

Finally, we preregistered that, if both gendered learning affordances and genetic advantages emerged as significant predictors of the observed gender difference in Study 1, we would test these two variables as competing predictors. Since we found support for both predictors among systemizing activities, we tested a model where gendered learning affordances and genetic advantages were entered as simultaneous predictors of the observed gender difference on systemizing items only in Study 1.^13^ The two predictors were moderately correlated, *r*(23) *=* .56, *p =* .003. Perceptions of having gendered learning affordances emerged as a significant predictor (*β* = .57, *p* = .003, 95% CI [.21, .93]), whereas the relationship for perceived genetic advantage became nonsignificant (*β* = .25, *p* = .158, 95% CI [−.11, .61]).^14^

### Discussion

As a preregistered replication and extension of Study 2, the results of Study 3 provide a stronger test of the degree to which measures of systemizing and empathizing assess gender differences that are perceived to reflect learning affordances. Like lay coders, experts rated systemizing and empathizing activities as more likely to be learned through experience, rather than innate abilities. Notably, experts believed that men have significantly more affordances to learn every systemizing activity, and unlike in Study 2, they also indicated that women have significantly more affordances to learn empathizing activities. Supporting our preregistered hypothesis and replicating Study 2, activities in which experts rate men and women as having different affordances are also the activities that show the largest gender differences in Study 1. For systemizing, perceived differences in affordances predicted observed differences more than perceived genetic advantages. As hypothesized, experts were able to accurately estimate which systemizing and empathizing activities show the greatest gender differences.

One unexpected finding in our data is that empathizing activities coders rated as being relatively less learned through experience were also those that women tended to score higher on. Although suggestive of a somewhat more biologically based theory of empathizing, the interaction testing the difference in slopes was only marginally significant and expert ratings of women’s genetic advantages on empathizing activities did not predict gender differences. Given these mixed findings, we hesitate to draw strong conclusions. However, assuming some signal in these findings, this discrepancy between the perceived origin of individual variability versus gender-based variability in these activities might be a fruitful topic for further research.

Taken together, the results of Study 3 suggest that large gender differences observed on the SQ- and EQ-Short scales are related to experts’ perceptions of men’s and women’s different affordances to learn activities referenced in the items. Importantly, there is little evidence that even highly established experts in the field believe most items on these scales capture skills that are more innate than learned. Although most experts likely endorse interactional influences on complex behavior, it is notable that each and every activity on the systemizing scale was seen as affording more learning opportunities to men. A belief that the SQ-Short measures an innate drive to construct and analyze systems typical of a “male brain” would seem to be out of step with a consensus view from psychological science.

## General Discussion

Gender differences measured using the SQ and EQ are often cited as evidence for innate sex differences in systemizing and empathizing (Archer, 2019), consistent with theorizing about essentialized male and female brains (Baron-Cohen, 2002, 2004, 2009). Although prior revisions have attempted to debias these measures, research has not addressed whether the activities assessed in these scales are perceived to be valid indicators of gender differences in innate abilities or socially learned preferences. Accordingly, this work set out to address two questions:

**Research Question 1:** Do the SQ- and EQ-Short ask about activities perceived to reflect men's and women’s innate differences and/or different learning affordances?

**Research Question 2:** Do perceived innate differences or learning affordances better predict the size of the gender difference observed on the SQ- and EQ-Short items?

First, the items used to measure systemizing are especially geared toward activities perceived as learnable and for which men are seen as having a greater opportunity to learn. Both lay coders and experts rated the majority (96% in Study 2 and 80% in Study 3) of systemizing activities as being more reflective of interests that are learned through experience than innate ability. They also consistently estimated that men have significantly more affordances to learn systemizing activities. Experts in Study 3, but not lay perceivers in Study 2, perceived that women have more affordances to learn empathizing activities.

Turning to the relationship between perceived learning affordances and observed gender differences, results supported preregistered predictions. Activities that expert (and lay) coders assume men have more affordances to learn were the items men scored higher on in Study 1. These effects were sometimes stronger for systemizing items and replicated when we analyzed data by subdisciplines (see SOM). Importantly, there was no evidence that experts or lay coders believe sex-based genetic advantages better explain observed differences in systemizing and empathizing. Together these results suggest that the gender difference on self-report measures of systemizing (and to a lesser degree, empathizing) can be said to reflect a consensus perception of men and women’s different opportunities to learn these activities, perhaps more than perceived genetic advantages.

It is unclear why the effects in Study 3, in particular, were stronger for systemizing than empathizing. In this study, experts’ perceived learning affordances, relative to their perceived genetic advantages, were stronger predictors of gender differences measured in the target sample, but the effect was weaker for empathizing than for systemizing items. The greater specificity of tasks on the systemizing scale might have made it easier for experts, in particular, to ascertain situational affordances for learning these discrete skills. However, given that this same pattern was not found in Study 2, we hesitate to draw firm conclusions.

### Implications

E-S theory’s claims of male and female brains have far-reaching consequences outside of the ivory tower. Claims about inherent gender differences in systemizing and empathizing have the potential to fuel gender discrimination, undermine women’s sense of fit and belonging, and guide gendered career selections. Given these repercussions, a responsible science must closely examine the evidence used to support these claims.

Our results suggest measurement of systemizing might be biased by activities that men are seen as having more opportunities than women to learn. Similar, albeit weaker, effects were observed for the measurement of empathizing. Our work extends prior efforts to debias the SQ (i.e., adding systemizing items in feminine domains in the SQ-R, Wheelwright et al., 2006; removing items that function differently by gender, Allison et al., 2015) by empirically pinpointing perceived affordances in SQ and EQ items as a possibly contributing to the size of the observed gender difference. A self-report measure that is not biased by perceived affordances would need to assess systemizing entirely in the context of activities that men and women have relatively equal opportunities to learn. Similar efforts have been taken in the development of career interests (Su et al., 2009). Until that time, measured effect sizes on the SQ in particular should not be interpreted as evidence of innate sex differences.

Beyond informing the SQ and EQ measures, our findings illuminate areas of practical focus for researchers. Subsequent efforts to develop self-report measures might incorporate a step in the validation process that aims to equate items on the degree to which they provide learning affordances across genders. Possible strategies to this end could include: (a) reflecting on one’s own positionality during item generation, (b) crowdsourcing wisdom from diverse groups to yield a large pool of potential items, and/or (c) using more abstract items that focus on the process rather than specific activities tied to learning affordances. Most importantly, one must recognize that observed differences between men and women on any self-report measure cannot provide clear evidence of the origin of those differences.

### Limitations and Future Directions

#### Difficulty in Determining Etiology

One key limitation is the difficulty of determining the true etiology of gender differences on any construct; indeed, this goal is beyond the scope of our paper. As our goal was to assess the legitimacy of claims that the SQ and EQ could assess innate sex differences, we asked coders to consider only the perceived relative strength of genetic and environmental affordances and did not ask about more complex causal forces. Although the wisdom of crowds approach suggests that diverse and expert coders can accurately estimate the true effects of gender differences, their causal explanations for these estimates might still be biased by coders’ own point of view. For example, even experts in human behavior had varied perceptions of the causal forces at work as suggested by the evidence that expert coders’ explanations for gender differences (whether learned or genetic) differed by subdiscipline in Study 3 (see SOM). Our wisdom of crowds approach was designed to balance out these individual biases and errors, but we acknowledge such estimates cannot be assumed to reflect what is likely a complex set of processes shaping people’s true interests and abilities. Future research could perhaps consider more complex understandings, for instance, by exploring interactional effects between environment and biology.

#### Constraints on Generalizability

To bolster the replicability of our work, we acknowledge the contextual and population-level factors that likely present boundary conditions for our work (Simons et al., 2017). First, given that gender stereotypes tend to change over time (Charlesworth & Banaji, 2022) and are situated within culture (Miller et al., 2015), we might not expect the content of ratings (i.e., estimated gender differences on specific activities) to replicate beyond this time period nor cultural context. Another boundary condition presented by our work is our inability to speak to these effects as they apply beyond gender as a single-axis identity. Work on intersectionality reveals that gendered phenomena often vary across the intersection of race, class, sexual orientation, and other social identities (Crenshaw, 1989; Petsko et al., 2022). In addition, as our focus is on men and women, we are unable to speak to whether effects would replicate beyond these binary gender identities (Morgenroth & Ryan, 2018). Scholars might consider these boundary conditions as important directions for further work on this topic.

#### Alternative Sources of Gender Bias in the Activities

Our work draws attention to the possibility that gender differences in EQ and SQ activities are best understood as reflecting different learning opportunities experienced by men and women. This provokes a further question of whether these effects are more strongly driven by prescriptive norms, things that society generally believes men and women ought to be (Rudman & Glick, 2001), or proscriptive norms, things that society generally believes men and women ought *not* to be (Vandello & Bosson, 2013). For example, because of strong gender stereotypes about women’s emotional and communal nature (Brescoll, 2016; Eagly et al., 2020; Shields, 2002): (a) women might be encouraged to learn these empathizing activities via prescriptive norms and/or (b) men might be actively discouraged from learning these same activities via proscriptive norms. Future research might work to disentangle these distinct contributors.

## Conclusion

The SQ and EQ self-report measures have been employed for nearly two decades to provide evidence for biologically based accounts of sex differences on systemizing and empathizing. Although prior revisions to the SQ and EQ have addressed sources of gender bias in the measures, the present findings make a unique contribution by suggesting that gender differences on the SQ and EQ are correlated with the different learning affordances men are women are assumed to have. By better understanding perceived affordances as a potential source of gender bias in self-report measures of systemizing and (to a lesser degree) empathizing, researchers may move toward a more complete understanding of these constructs and how best to measure and interpret them.

## Supplemental Material

sj-docx-1-psp-10.1177_01461672231202268 – Supplemental material for Do Measures of Systemizing and Empathizing Reflect Perceptions of Gender Differences in Learning Affordances? Supplemental material, sj-docx-1-psp-10.1177_01461672231202268 for Do Measures of Systemizing and Empathizing Reflect Perceptions of Gender Differences in Learning Affordances? by Audrey Aday, Toni Schmader and Michelle Ryan in Personality and Social Psychology Bulletin

## Notes

1. We use the term *gender differences* to refer to observed differences between those who self-identify as women or men, acknowledging this distinction does not capture identities beyond these binary categories. We use *sex differences* when referring to what are assumed to be essentialized differences based on sex assigned at birth.

2. The original SQ- and EQ-Short included four forced-choice response options, treated all “disagree” options as equivalent, and summed rather than averaged scores (see Wakabayashi et al., 2006). However, including more scale points and a “Neutral” response option reduces response bias and improves reliability (see Croasmun & Ostrom, 2011).

3. These analyses do not include *N =* 2 participants who identified as nonbinary and *N =* 5 participants who did not report their gender.

4. There were no items that showed an effect size of exactly *d =* .30; thus, all items were binned into one of the three predefined categories.

5. There was a technical error that affected four of the 100 ratings (see SOM). Reanalyzing data excluding these ratings only changed one result: the interaction between genetic differences and item type changes from marginal (*β* = .27, *p* = .054) to significant (*β* = .31, *p* = .033).

6. Using the unweighted mean is a common estimate of wisdom of crowds (Lorenz et al., 2011), though some studies (e.g., Sjöberg, 2009) use median coder ratings. In these data, several ratings showed a median of 4, resulting in low or zero variance. To maximize variance, we use the unweighted mean. In addition, we did not analyze data in an MLM framework since our goal was to model aggregate item-level information. Results are unchanged by this decision unless noted otherwise. We report all effects by coder gender and modeled in MLM in Supplemental Appendix A.

7. For item-level comparisons reported in Studies 2 and 3, we report Welch’s *t*-test (Welch, 1947), which is more robust against Type I Error than Student’s *t*-test when variables have unequal variance and/or unequal sample sizes (Delacre et al., 2017). To compute degrees of freedom, Welch’s *t*-test uses the Satterthwaite–Welch adjustment (Satterthwaite, 1946).

8. Exploratory simple slope analyses revealed this pattern was largely driven by empathizing activities, *β* = 0.51, *p* = .038, and not systemizing activities, *β* = 0.10, *p* = .308. Simple slopes by empathizing and systemizing for all regression analyses reported in Studies 2 and 3 are included in the SOM.

9. In *N =* 2 cases, participants’ self-identified gender was not the same gender recorded in our recruitment list and we defaulted to participants’ self-identified gender.

10. We preregistered we would test hypotheses with MLM if there was substantial dependency within subdiscipline or coder gender. As specified in Study 2, we later reasoned it was more sensible to model the unweighted item-level mean. Effects are unchanged by this decision unless noted otherwise. We report all effects by coder gender, by subdiscipline, and modeled in MLM in the SOM.

11. When modeled with MLM, the main effect of coder ratings on the observed gender difference is significant (see SOM).

12. Preregistered simple slopes analyses showed the relationship between expert ratings and observed gender differences was not significant for systemizing (*β* = .01, *p* = .872) and significant in the opposite expected direction for empathizing (*β* = .39, *p* = .042). As in Study 2, empathizing activities that experts judge to be less learned through experience were those that women scored higher on.

13. Although we preregistered modeling all items with subscale as a moderating factor, analyses revealed item-level average ratings of gendered and genetic advantage were highly correlated, *r*(45) = .93. Since the systemizing items showed a more reasonable correlation magnitude and demonstrate the strongest effects, we report these analyses. Results are robust to either methodological decision.

14. When modeled in MLM, although the ratio of magnitude between predictors is similar (gendered learning affordances: *b =* .07, *p* < .001; genetic advantage: *b =* .03, *p = .*013), genetic advantage also emerged as a significant predictor.

## References

[bibr1-01461672231202268] Aday A. (2023). Just not that interested? Drivers of the gender gap on systemizing and empathizing interest [Doctoral dissertation]. University of British Columbia.

[bibr2-01461672231202268] Allison C. Baron-Cohen S. Stone M. H. Muncer S. J. (2015). Rasch modeling and confirmatory factor analysis of the Systemizing Quotient-Revised (SQ-R) Scale. The Spanish Journal of Psychology, 18, E16. 10.1017/sjp.2015.19 25818099

[bibr3-01461672231202268] Allison C. Baron-Cohen S. Wheelwright S. J. Stone M. H. Muncer S. J. (2011). Psychometric analysis of the Empathy Quotient (EQ). Personality and Individual Differences, 51(7), 829–835.

[bibr4-01461672231202268] Archer J. (2019). The reality and evolutionary significance of human psychological sex differences. Biological Reviews, 94(4), 1381–1415. 30892813 10.1111/brv.12507

[bibr5-01461672231202268] Auyeung B. Wheelwright S. Allison C. Atkinson M. Samarawickrema N. Baron-Cohen S. (2009). The children’s empathy quotient and systemizing quotient: Sex differences in typical development and in autism spectrum conditions. Journal of Autism and Developmental Disorders, 39(11), 1509–1521. 19533317 10.1007/s10803-009-0772-x

[bibr6-01461672231202268] Baron-Cohen S. (2002). The extreme male brain theory of autism. Trends in Cognitive Sciences, 6(6), 248–254. 12039606 10.1016/s1364-6613(02)01904-6

[bibr7-01461672231202268] Baron-Cohen S. (2004). The essential difference: Men, women, and the extreme male brain. Penguin.

[bibr8-01461672231202268] Baron-Cohen S. (2009). Autism: The empathizing–systemizing (E-S) theory. Annals of the New York Academy of Sciences, 1156(1), 68–80. 19338503 10.1111/j.1749-6632.2009.04467.x

[bibr9-01461672231202268] Baron-Cohen S. Richler J. Bisarya D. Gurunathan N. Wheelwright S. (2003). The systemizing quotient: An investigation of adults with Asperger syndrome or high–functioning autism, and normal sex differences. Philosophical Transactions of the Royal Society of London, Series B: Biological Sciences, 358(1430), 361–374. 12639333 10.1098/rstb.2002.1206 PMC1693117

[bibr10-01461672231202268] Baron-Cohen S. Wheelwright S. (2004). The empathy quotient: An investigation of adults with Asperger syndrome or high functioning autism, and normal sex differences. Journal of Autism and Developmental Disorders, 34(2), 163–175. 15162935 10.1023/b:jadd.0000022607.19833.00

[bibr11-01461672231202268] Baron-Cohen S. Wheelwright S. Hill J. Raste Y. Plumb I. (2001). The “Reading the Mind in the Eyes” Test revised version: A study with normal adults, and adults with Asperger syndrome or high-functioning autism. The Journal of Child Psychology and Psychiatry and Allied Disciplines, 42(2), 241–251. 11280420

[bibr12-01461672231202268] Bian L. Leslie S. J. Murphy M. C. Cimpian A. (2018). Messages about brilliance undermine women’s interest in educational and professional opportunities. Journal of Experimental Social Psychology, 76, 404–420.

[bibr13-01461672231202268] Brescoll V. L. (2016). Leading with their hearts? How gender stereotypes of emotion lead to biased evaluations of female leaders. The Leadership Quarterly, 27(3), 415–428.

[bibr14-01461672231202268] Butler J. (1990). Gender trouble: Feminism and the subversion of identity. Routledge.

[bibr15-01461672231202268] Chapman E. Baron-Cohen S. Auyeung B. Knickmeyer R. Taylor K. Hackett G. (2006). Fetal testosterone and empathy: Evidence from the empathy quotient (EQ) and the “reading the mind in the eyes” test. Social Neuroscience, 1(2), 135–148. 18633782 10.1080/17470910600992239

[bibr16-01461672231202268] Charlesworth T. E. Banaji M. R. (2022). Patterns of implicit and explicit stereotypes III: Long-term change in gender stereotypes. Social Psychological and Personality Science, 13(1), 14–26.

[bibr17-01461672231202268] Cheryan S. Markus H. R. (2020). Masculine defaults: Identifying and mitigating hidden cultural biases. Psychological Review, 127(6), 1022. 32804526 10.1037/rev0000209

[bibr18-01461672231202268] Cowansage C. (2017, August). Ask a female engineer: Thoughts on the Google memo. https://www.ycombinator.com/blog/ask-a-female-engineer-thoughts-on-the-google-memo

[bibr19-01461672231202268] Crenshaw K. (1989). Demarginalizing the intersection of race and sex: A Black feminist critique of antidiscrimination doctrine, feminist theory and antiracist politics (pp. 139–168). The University of Chicago legal forum.

[bibr20-01461672231202268] Croasmun J. T. Ostrom L. (2011). Using Likert-type scales in the social sciences. Journal of Adult Education, 40(1), 19–22.

[bibr21-01461672231202268] Damore J. (2017, July). Google’s ideological echo chamber: How bias clouds our thinking about diversity and inclusion. https://s3.documentcloud.org/documents/3914586/Googles-Ideological-Echo-Chamber.pdf

[bibr22-01461672231202268] Dar-Nimrod I. Heine S. J. (2006). Exposure to scientific theories affects women’s math performance. Science, 314(5798), 435. 17053140 10.1126/science.1131100

[bibr23-01461672231202268] Deaux K. Major B. (1987). Putting gender into context: An interactive model of gender-related behavior. Psychological Review, 94(3), 369–389.

[bibr24-01461672231202268] Delacre M. Lakens D. Leys C. (2017). Why psychologists should by default use Welch’s t-test instead of Student’s t-test. International Review of Social Psychology, 30(1), 92–101.

[bibr25-01461672231202268] Eagly A. H. Nater C. Miller D. I. Kaufmann M. Sczesny S. (2020). Gender stereotypes have changed: A cross-temporal meta-analysis of US public opinion polls from 1946 to 2018. American Psychologist, 75(3), 301–315. 31318237 10.1037/amp0000494

[bibr26-01461672231202268] Eagly A. H. Revelle W. (2022). Understanding the magnitude of psychological differences between women and men requires seeing the forest and the trees. Perspectives on Psychological Science, 17, 1339–1358. 35532752 10.1177/17456916211046006 PMC9442632

[bibr27-01461672231202268] Eagly A. H. Wood W. (2012). Social role theory. In Van Lange P. Higgins T. Kruglanski A. (Eds.), Handbook of theories of social psychology (pp. 458–476). Sage.

[bibr28-01461672231202268] Eccles J. S. Freedman-Doan C. Frome P. Jacobs J. Yoon K.S. (2000). Gender-role socialization in the family: A longitudinal approach. In Eckes T. Trautner H. M. (Eds.), The developmental social psychology of gender (pp. 333–360). Lawrence Erlbaum Associates Publishers.

[bibr29-01461672231202268] Fine C. (2010). Delusions of gender: How our minds, society, and neurosexism create difference. W.W. Norton.

[bibr30-01461672231202268] Fine C. (2012). Explaining, or sustaining, the status quo? The potentially self-fulfilling effects of “hardwired” accounts of sex differences. Neuroethics, 5(3), 285–294.

[bibr31-01461672231202268] Gillis-Buck E. M. Richardson S. S. (2014). Autism as a biomedical platform for sex differences research. BioSocieties, 9(3), 262–283.

[bibr32-01461672231202268] Grossi G. Fine C. (2012). The role of fetal testosterone in the development of the “essential difference” between the sexes: Some essential issues. In Bluhm R. Jacobson A. J. Maibom H. L. (eds) Neurofeminism: New directions in philosophy and cognitive science (pp. 73–104). Palgrave Macmillan.

[bibr33-01461672231202268] Harrison J. L. Brownlow C. L. Ireland M. J. Piovesana A. M. (2022). Empathy measurement in autistic and nonautistic adults: A COSMIN systematic literature review. Assessment, 29(2), 332–350. 33070621 10.1177/1073191120964564

[bibr34-01461672231202268] Hyde J. S. (2014). Gender similarities and differences. Annual Review of Psychology, 65, 373–398. 10.1146/annurev-psych-010213-115057 23808917

[bibr35-01461672231202268] Hyde J. S. Bigler R. S. Joel D. Tate C. C. van Anders S. M. (2019). The future of sex and gender in psychology: Five challenges to the gender binary. American Psychologist, 74(2), 171–193. 30024214 10.1037/amp0000307

[bibr36-01461672231202268] Joel D. Berman Z. Tavor I. Wexler N. Gaber O. Stein Y. Shefi N. Pool J. Urchs S. Margulies D. S. Liem F. Hänggi J. Jäncke L. Assaf Y. (2015). Sex beyond the genitalia: The human brain mosaic. Proceedings of the National Academy of Sciences of the United States of America, 112(50), 15468–15473. 26621705 10.1073/pnas.1509654112 PMC4687544

[bibr37-01461672231202268] Kirkland R. A. Peterson E. Baker C. A. Miller S. Pulos S. (2013). Meta-analysis reveals adult female superiority in “reading the mind in the eyes test.” North American Journal of Psychology, 15(1), 121–146.

[bibr38-01461672231202268] Koo T. K. Li M. Y. (2016). A guideline of selecting and reporting intraclass correlation coefficients for reliability research. Journal of Chiropractic Medicine, 15(2), 155–163. 27330520 10.1016/j.jcm.2016.02.012 PMC4913118

[bibr39-01461672231202268] Kühnen U. Hannover B. Roeder U. Shah A. A. Schubert B. Upmeyer A. Zakaria S. (2001). Cross-cultural variations in identifying embedded figures: Comparisons from the United States, Germany, Russia, and Malaysia. Journal of Cross-Cultural Psychology, 32(3), 366–372.

[bibr40-01461672231202268] Larrick R. P. Mannes A. E. Soll J. B. (2011). The social psychology of the wisdom of crowds. In Krueger J. I. (Ed.), Frontiers in social psychology: Social judgment and decision making (pp. 227–242). Psychology Press.

[bibr41-01461672231202268] Lorenz J. Rauhut H. Schweitzer F. Helbing D. (2011). How social influence can undermine the wisdom of crowd effect. Proceedings of the National Academy of Sciences, 108(22), 9020–9025. 10.1073/pnas.1008636108 PMC3107299 21576485

[bibr42-01461672231202268] Lytton H. Romney D. M. (1991). Parents’ differential socialization of boys and girls: A meta-analysis. Psychological Bulletin, 109(2), 267–296.

[bibr43-01461672231202268] Manning J. T. Baron-Cohen S. Wheelwright S. Fink B. (2010). Is digit ratio (2D: 4D) related to systemizing and empathizing? Evidence from direct finger measurements reported in the BBC internet survey. Personality and Individual Differences, 48(6), 767–771.

[bibr44-01461672231202268] Miller D. I. Eagly A. H. Linn M. C. (2015). Women’s representation in science predicts national gender-science stereotypes: Evidence from 66 nations. Journal of Educational Psychology, 107(3), 631–644.

[bibr45-01461672231202268] Morgenroth T. Ryan M. K. (2018). Gender trouble in social psychology: How can Butler’s work inform experimental social psychologists’ conceptualization of gender? Frontiers in Psychology, 9, Article 1320. 10.3389/fpsyg.2018.01320 PMC6072877 30100895

[bibr46-01461672231202268] Muncer S. J. Ling J. (2006). Psychometric analysis of the Empathy Quotient (EQ) Scale. Personality and Individual Differences, 40(6), 1111–1119.

[bibr47-01461672231202268] O’Connor C. Joffe H. (2020). Intercoder reliability in qualitative research: Debates and practical guidelines. International Journal of Qualitative Methods, 19, 1609406919899220.

[bibr48-01461672231202268] Oppenheimer D. M. Meyvis T. Davidenko N. (2009). Instructional manipulation checks: Detecting satisficing to increase statistical power. Journal of Experimental Social Psychology, 45(4), 867–872.

[bibr49-01461672231202268] Petsko C. D. Rosette A. S. Bodenhausen G. V. (2022). Through the looking glass: A lens-based account of intersectional stereotyping. Journal of Personality and Social Psychology, 123(4), 763–787. 35025602 10.1037/pspi0000382

[bibr50-01461672231202268] Rudman L. A. Glick P. (2001). Prescriptive gender stereotypes and backlash toward agentic women. Journal of Social Issues, 57(4), 743–762.

[bibr51-01461672231202268] Satterthwaite F. E. (1946). An approximate distribution of estimates of variance components. Biometrics Bulletin, 2(6), 110–114. 20287815

[bibr52-01461672231202268] Schroeter M. L. Kynast J. Schlögl H. Baron-Cohen S. Villringer A. (2022). Sex and age interact in reading the mind in the eyes. Comprehensive Psychoneuroendocrinology, 12, 100162. 36411783 10.1016/j.cpnec.2022.100162 PMC9674865

[bibr53-01461672231202268] Shields S. A. (2002). Speaking from the heart: Gender and the social meaning of emotion. Cambridge University Press.

[bibr54-01461672231202268] Shrout P. E. Fleiss J. L. (1979). Intraclass correlations: Uses in assessing rater reliability. Psychological Bulletin, 86(2), 420–428. 18839484 10.1037//0033-2909.86.2.420

[bibr55-01461672231202268] Simons D. J. Shoda Y. Lindsay D. S. (2017). Constraints on generality (COG): A proposed addition to all empirical papers. Perspectives on Psychological Science, 12(6), 1123–1128. 28853993 10.1177/1745691617708630

[bibr56-01461672231202268] Sjöberg L. (2009). Are all crowds equally wise? A comparison of political election forecasts by experts and the public. Journal of Forecasting, 28(1), 1–18.

[bibr57-01461672231202268] Su R. Rounds J. Armstrong P. I. (2009). Men and things, women and people: A meta-analysis of sex differences in interests. Psychological Bulletin, 135(6), 859–884. 19883140 10.1037/a0017364

[bibr58-01461672231202268] Swim J. K. (1994). Perceived versus meta-analytic effect sizes: An assessment of the accuracy of gender stereotypes. Journal of Personality and Social Psychology, 66(1), 21–36.

[bibr59-01461672231202268] Tenenbaum H. R. Leaper C. (2003). Parent-child conversations about science: The socialization of gender inequities? Developmental Psychology, 39(1), 34–47. 12518807 10.1037//0012-1649.39.1.34

[bibr60-01461672231202268] Vandello J. A. Bosson J. K. (2013). Hard won and easily lost: A review and synthesis of theory and research on precarious manhood. Psychology of Men & Masculinity, 14(2), 101–113.

[bibr61-01461672231202268] Wakabayashi A. Baron-Cohen S. Wheelwright S. Goldenfeld N. Delaney J. Fine D. . . .Weil L. (2006). Development of short forms of the Empathy Quotient (EQ-Short) and the Systemizing Quotient (SQ-Short). Personality and Individual Differences, 41(5), 929–940.

[bibr62-01461672231202268] Welch B. L. (1947). The generalization of ‘Student’s’ problem when several different population variances are involved. Biometrika, 34(1–2), 28–35. 20287819 10.1093/biomet/34.1-2.28

[bibr63-01461672231202268] Wheelwright S. Baron-Cohen S. Goldenfeld N. Delaney J. Fine D. Smith R. . . . Wakabayashi A. (2006). Predicting Autism Spectrum Quotient (AQ) from the Systemizing Quotient-Revised (SQ-R) and Empathy Quotient (EQ). Brain Research, 1079(1), 47–56. 16473340 10.1016/j.brainres.2006.01.012

[bibr64-01461672231202268] Witkin H. A. Oltman P. K. Raskin E. Karp S. A. (1971). A manual for the embedded-figures tests. Consulting Psychologists Press.

[bibr65-01461672231202268] Zell E. Krizan Z. Teeter S. R. (2015). Evaluating gender similarities and differences using metasynthesis. American Psychologist, 70(1), 10–20. 25581005 10.1037/a0038208

