# Supplementary material for: Do Measures of Systemizing and Empathizing Reflect Perceptions of Gender Differences in Learning Affordances?
Source: Pers Soc Psychol Bull. 2023 Oct 21;51(5):845–62. doi: 10.1177/01461672231202268 (PMC11930639; doi:10.1177/01461672231202268)
Supplement: sj-docx-1-psp-10.1177_01461672231202268 – Supplemental material for Do Measures of Systemizing and Empathizing Reflect Perceptions of Gender Differences in Learning Affordances? [file sj-docx-1-psp-10.1177_01461672231202268.docx]

# Supplementary Online Materials

This section contains supplementary materials for “Do Measures of Systemizing and Empathizing Reflect Perceptions of Gender Differences in Learning Affordances?”

**Study 1: Estimating SQ and EQ Gender Differences**

*Study 1 - List of Measures*

Below is the full list of variables included in Study 1 in the order they were presented to participants. Variables analyzed and reported in the main text are denoted with an asterisk.

- Attention Check
- SQ-Short* / EQ-Short*
- Gender*
- Age*
- Ethnic/Racial Background*
- Region*
- Political Orientation*
- SES

*Study 1 -* *Items for Measures*

**Attention Check**

Research in decision making shows that people, when making decisions and answering questions, prefer not to pay attention and minimize their effort as much as possible. Some studies show that over 50% of people don’t carefully read questions. If you are reading this question and have read all the other questions, please select the box marked ‘other’ and type ‘Decision Making’ in the box below. Do not select “predictions of your own behavior.” Thank you for participating and taking the time to read through the questions carefully!

What is this study about?

- Predictions of your own behavior
- Predictions of your friend’s behavior
- Political preferences
- Other: _______________

**SQ-Short**

*1 = Strongly disagree . . . 7 = Strongly agree*

*(R) =* Reverse-scored

- 1. If I were buying a car, I would want to obtain specific information about its engine capacity.
- 2. If there was a problem with the electrical wiring in my home, I'd be able to fix it myself.
- 3. I rarely read articles or web pages about new technology. *(R)*
- 4. I do not enjoy games that involve a high degree of strategy. *(R)*
- 5. I am fascinated by how machines work.
- 6. In math, I am intrigued by the rules and patterns governing numbers.
- 7. I find it difficult to understand instruction manuals for putting appliances together. *(R)*
- 8. If I were buying a computer, I would want to know exact details about its hard disk drive capacity and processor speed.
- 9. I find it difficult to read and understand maps. *(R)*
- 10. When I look at a piece of furniture, I do not notice the details of how it was constructed.
- 11. I find it difficult to learn my way around a new city. *(R)*
- 12. I do not tend to watch science documentaries on television or read articles about science and nature. *(R)*
- 13. If I were buying a stereo, I would want to know about its precise technical features.
- 14. I find it easy to grasp exactly how odds work in betting.
- 15. I am not very meticulous when I carry out D.I.Y.
- 16. When I look at a building, I am curious about the precise way it was constructed.
- 17. I find it difficult to understand information the bank sends me on different investment and saving systems.
- 18. When traveling by train, I often wonder exactly how the rail networks are coordinated.
- 19. If I were buying a camera, I would not look carefully into the quality of the lens.
- 20. When I hear about the weather forecast, I am not very interested in the meteorological patterns.
- 21. When I look at a mountain, I think about how precisely it was formed.
- 22. I can easily visualize how the motorways in my region link up.
- 23. When I'm in a plane, I do not think about the aerodynamics. *(R)*
- 24. I am interested in knowing the path a river takes from its source to the sea.
- 25. I am not interested in understanding how wireless communication works. *(R)*

**EQ-Short**

​​*1 = Strongly disagree . . . 7 = Strongly agree*

*(R) =* Reverse-scored

- 1. I can easily tell if someone else wants to enter a conversation.
- 2. I really enjoy caring for other people.
- 3. I find it hard to know what to do in a social situation. *(R)*
- 4. I often find it difficult to judge if something is rude or polite. *(R)*
- 5. In a conversation, I tend to focus on my own thoughts rather than on what my listener might be thinking. *(R)*
- 6. I can pick up quickly if someone says one thing but means another.
- 7. It is hard for me to see why some things upset people so much. *(R)*
- 8. I find it easy to put myself in somebody else's shoes.
- 9. I am good at predicting how someone will feel.
- 10. I am quick to spot when someone in a group is feeling awkward or uncomfortable.
- 11. I can't always see why someone should have felt offended by a remark. *(R)*
- 12. I don't tend to find social situations confusing.
- 13. Other people tell me I am good at understanding how they are feeling and what they are thinking.
- 14. I can easily tell if someone else is interested or bored with what I am saying.
- 15. Friends usually talk to me about their problems as they say that I am very understanding.
- 16. I can sense if I am intruding, even if the other person doesn't tell me.
- 17. Other people often say that I am insensitive, though I don't always see why. *(R)*
- 18. I can tune into how someone else feels rapidly and intuitively.
- 19. I can easily work out what another person might want to talk about.
- 20. I can tell if someone is masking their true emotion.
- 21. I am good at predicting what someone will do.
- 22. I tend to get emotionally involved with a friend's problems.

*Study 1 – Sensitivity Analysis*

A sensitivity analysis performed in G*Power to detect a difference between two independent means (two groups) revealed that our sample of *N* = 624 could detect a *d* score of .23 or greater with 80% power, alpha = .05. Given that the original SQ- and EQ-Short validation paper (Wakabayashi et al., 2006) reported effect sizes of *d =* .95 and *d =* -.63 for gender differences on the SQ and EQ respectively, this sample is well-powered to detect effects.

*Study 1 – Sample Comparison to Census Demographic Data*

Table S.1 contains a comparison of Study 1’s sample demographics to each country’s (US, UK) census data. As seen below, our US and UK sample characteristics are fairly representative of key population level characteristics in these two countries from demographic census data taken near to the year when our data were collected. The only deviation is that our US sample underrepresents those who self-identify as Hispanic/Latinx likely because Prolific’s ethnicity question only included the response options: White, Mixed, Asian, Black, and Other. In addition, our sample is slightly older than the population given the exclusion of minors from research..

**Table S.1. Comparison of demographic characteristics of Study 1 samples from the US and UK to each country's census data.**

| **Gender** | **US Sample**  *N =* 313 | **US 2020**  **Census Data** | **UK Sample**  *N =* 306 | **UK 2021**  **Census Data** |
| --- | --- | --- | --- | --- |
| Man | 48.56% | 49.5% | 49.02% | 49% |
| Woman | 50.80% | 50.5% | 50.98% | 51% |
| Non-binary | 0.64% | no data | 0% | no data |
| **Racial/Ethnic Background** | **US Sample**  *N =* 313 | **US 2020**  **Census Data** | **UK Sample**  *N =* 306 | **UK 2021**  **Census Data** |
| Black | 13.74% | 13.6% | 5.23% | 4.04% |
| Asian | 7.98% | 6.1% | 9.48% | 9.25% |
| Hispanic or Latinx^1^ | 3.83% | 18.9% | 0.33% | no data |
| Indigenous | 0.96% | 1.6% | 0% | no data |
| Middle Eastern or Arabic | 0.32% | no data | 0.65% | 0.56% |
| White | 66.45% | 59.3% | 77.45% | 81.71% |
| Not Listed | 0.96% | no data | 6.21% | 1.55% |
| Multiracial | 5.75% | 2.9% | 0.65% | 2.88% |
| **Age** | **US Sample**  *N =* 313  *M* (SD) | **US 2020**  **Census Data** | **UK Sample**  *N =* 306  *M* (SD) | **UK 2021**  **Census Data** |
|  | 45.53 (16.10)  Md = 45 | Md = 38.5 | 45.23 (15.45)  Md = 46 | Md = 40.7 |

**^1^** Note that Prolific’s ethnicity question included the response options: White, Mixed, Asian, Black, and Other; it did not include a category for Hispanic/Latinx. Also, the samples are somewhat older on average given the omission of minors from research.

**Study 2**

*Study 2 -* *List of Measures*

Below is the full list of variables included in Study 2 in the order they were presented to participants. Variables analyzed and reported in the main text are denoted with an asterisk.

- Attention Check
- Learned vs. Innate Attributions*
- Estimated Gender Difference*
- The order of the following variables was counterbalanced:
  - Gendered Learning Affordances*
  - Genetic Differences*
- Fixed vs. Growth Mindset
- Gender*
- Age*
- Ethnic/Racial Background*
- Political Orientation*
- SES*

*Study 2 -* *Items for Measures*

**Attention Check**

Research in decision making shows that people, when making decisions and answering questions, prefer not to pay attention and minimize their effort as much as possible. Some studies show that over 50% of people don’t carefully read questions. If you are reading this question and have read all the other questions, please select the box marked ‘other’ and type ‘Decision Making’ in the box below. Do not select “predictions of your own behavior.” Thank you for participating and taking the time to read through the questions carefully!

What is this study about?

- Predictions of your own behavior
- Predictions of your friend’s behavior
- Political preferences
- Other: _______________

**SQ Activities**

- 1. Obtaining specific information about a car’s engine capacity.
- 2. Fixing a problem with the electrical wiring in one’s home.
- 3. Reading articles or web pages about new technology.
- 4. Enjoying games that involve a high degree of strategy.
- 5. Being fascinated by how machines work.
- 6. Being intrigued by the rules and patterns governing numbers in math.
- 7. Understanding instruction manuals for putting appliances together.
- 8. Knowing exact details about a computer’s hard disk drive capacity and processor speed.
- 9. Reading and understanding maps.
- 10. Noticing the details of how a piece of furniture was constructed.
- 11. Learning one’s way around a new city.
- 12. Watching science documentaries on television or reading articles about science and nature.
- 13. Knowing about a stereo’s precise technical features.
- 14. Grasping exactly how odds work in betting.
- 15. Being meticulous when carrying out D.I.Y.
- 16. Being curious about the precise way a building was constructed.
- 17. Understanding information the bank sends on different investment and saving systems.
- 18. Wondering exactly how rail networks of trains are coordinated.
- 19. Looking carefully into the quality of a camera lens.
- 20. Being interested in meteorological patterns.
- 21. Thinking about how precisely a mountain was formed.
- 22. Visualizing how the motorways in one’s region link up.
- 23. Thinking about the aerodynamics of a plane.
- 24. Knowing the path a river takes from its source to the sea.
- 25. Understanding how wireless communication works.

**EQ Activities**

*(R) =* Reverse-scored

Although not preregistered, we reverse-scored EQ Activities 5, 12, and 17 for directional ratings of the gender difference and gendered learning opportunities, since these activities are negatively worded (i.e., higher values corresponded with less empathizing). Not reverse-scoring these activities only changes 2 results: for empathizing items, (1) the relationship between estimate of gender difference and the size of the gender difference becomes non-significant, and (2) the relationship between gendered learning affordances and the size of the gender difference becomes marginal.

- 1. Telling if someone else wants to enter a conversation.
- 2. Caring for other people.
- 3. Knowing what to do in a social situation.
- 4. Judging if something is rude or polite.
- 5. Focusing on one’s own thoughts rather than on what their listener might be thinking. *(R)*
- 6. Picking up quickly if someone says one thing but means another.
- 7. Seeing why some things upset people so much.
- 8. Putting oneself in somebody else's shoes.
- 9. Predicting how someone will feel.
- 10. Spotting when someone in a group is feeling awkward or uncomfortable.
- 11. Seeing why someone should have felt offended by a remark.
- 12. Finding social situations confusing. *(R)*
- 13. Understanding how other people are feeling and what they are thinking.
- 14. Telling if someone else is interested or bored with what one is saying.
- 15. Being very understanding.
- 16. Sensing if one is intruding.
- 17. Being insensitive. *(R)*
- 18. Tuning into how someone else feels.
- 19. Working out what another person might want to talk about.
- 20. Telling if someone is masking their true emotion.
- 21. Predicting what someone will do.
- 22. Getting emotionally involved with a friend's problems.

*Study 2 – Technical Error Information*

In the survey for Study 2, there was a technical error where 3 EQ ratings (EMP 11, EMP 14, and EMP 19) contained the correct rating scale but the incorrect question stem. Additionally, there were minor display issues for a group of SQ ratings that did not affect the content of the ratings themselves and one case where the SQ rating labels were reversed for half of participants (SYS 7). As specified in the main text, reanalyzing our data excluding items affected by these technical errors only changes one result, such that the interaction between genetic differences and item type changes from marginal (𝛽 = .27, *p* = .054) to significant (𝛽 = .31, *p* = .033). All other results are unchanged.

*Study 2 – Item-Level Descriptive Statistics*

**Table S.2. Item-level information on coder ratings for each measure and the *p-*value for *t-*tests comparing ratings to scale midpoint (Study 2)**

| Variable | Item Text | Gender Differences | | | | Innate vs. Learned Attributions | | | | Gendered Learning Affordances | | | | Genetic Differences | | | |
| --- | --- | --- | --- | --- | --- | --- | --- | --- | --- | --- | --- | --- | --- | --- | --- | --- | --- |
|  |  | Mean | SD | Above MP  (*p*) | Below MP  (*p*) | Mean | SD | Above MP  (*p*) | Below MP  (*p*) | Mean | SD | Above MP  (*p*) | Below MP  (*p*) | Mean | SD | Above MP  (*p*) | Below MP  (*p*) |
| EMP1 | Telling if someone else wants to enter a conversation. | 3.89 | 1.60 | 0.696 | 0.304 | 4.57 | 1.49 | 0.004 | 0.996 | 4.11 | 1.44 | 0.284 | 0.716 | 3.98 | 1.85 | N/A | N/A |
| EMP10 | Spotting when someone in a group is feeling awkward or uncomfortable. | 3.47 | 1.38 | 0.996 | 0.004 | 4.38 | 1.90 | 0.077 | 0.923 | 3.96 | 1.51 | 0.572 | 0.428 | 3.58 | 1.76 | N/A | N/A |
| EMP11 | Seeing why someone should have felt offended by a remark. | 3.69 | 1.59 | 0.951 | 0.049 | 4.88 | 1.54 | 0.000 | 1.000 | 3.93 | 1.45 | 0.655 | 0.345 | 3.88 | 1.76 | N/A | N/A |
| EMP12 | Finding social situations confusing. | 4.33 | 1.49 | 0.058 | 0.942 | 4.15 | 1.91 | 0.282 | 0.718 | 4.24 | 1.50 | 0.135 | 0.865 | 3.67 | 1.91 | N/A | N/A |
| EMP13 | Understanding how other people are feeling and what they are thinking. | 3.47 | 1.55 | 0.998 | 0.002 | 4.47 | 1.70 | 0.010 | 0.990 | 3.74 | 1.63 | 0.910 | 0.090 | 4.18 | 1.72 | N/A | N/A |
| EMP14 | Telling if someone else is interested or bored with what one is saying. | 4.08 | 1.26 | 0.330 | 0.670 | 4.68 | 1.73 | 0.003 | 0.997 | 4.60 | 1.35 | 0.001 | 0.999 | 4.15 | 2.07 | N/A | N/A |
| EMP15 | Being very understanding. | 3.81 | 1.71 | 0.828 | 0.172 | 4.21 | 1.76 | 0.150 | 0.850 | 3.84 | 1.67 | 0.795 | 0.205 | 4.43 | 1.82 | N/A | N/A |
| EMP16 | Sensing if one is intruding. | 3.88 | 1.52 | 0.753 | 0.247 | 4.59 | 1.76 | 0.002 | 0.998 | 3.95 | 1.49 | 0.621 | 0.379 | 4.00 | 1.73 | N/A | N/A |
| EMP17 | Being insensitive. | 4.76 | 1.33 | 0.000 | 1.000 | 4.21 | 1.75 | 0.150 | 0.850 | 4.57 | 1.44 | 0.000 | 1.000 | 4.06 | 1.76 | N/A | N/A |
| EMP18 | Tuning into how someone else feels. | 3.66 | 1.78 | 0.951 | 0.049 | 4.19 | 1.91 | 0.197 | 0.803 | 4.01 | 1.57 | 0.471 | 0.529 | 4.39 | 1.71 | N/A | N/A |
| EMP19 | Working out what another person might want to talk about. | 4.04 | 1.34 | 0.418 | 0.582 | 4.61 | 1.47 | 0.002 | 0.998 | 4.37 | 1.25 | 0.019 | 0.981 | 4.37 | 1.57 | N/A | N/A |
| EMP2 | Caring for other people. | 3.50 | 1.63 | 0.995 | 0.005 | 4.00 | 1.78 | 0.500 | 0.500 | 3.59 | 1.78 | 0.976 | 0.024 | 4.28 | 1.85 | N/A | N/A |
| EMP20 | Telling if someone is masking their true emotion. | 3.97 | 1.57 | 0.558 | 0.442 | 4.76 | 1.73 | 0.000 | 1.000 | 4.18 | 1.49 | 0.143 | 0.857 | 4.17 | 1.81 | N/A | N/A |
| EMP21 | Predicting what someone will do. | 4.06 | 1.29 | 0.374 | 0.626 | 4.42 | 1.54 | 0.027 | 0.973 | 4.35 | 1.06 | 0.011 | 0.989 | 3.48 | 1.98 | N/A | N/A |
| EMP22 | Getting emotionally involved with a friend's problems. | 3.42 | 1.76 | 0.997 | 0.003 | 4.13 | 1.75 | 0.258 | 0.742 | 3.49 | 1.76 | 0.993 | 0.007 | 4.17 | 1.64 | N/A | N/A |
| EMP3 | Knowing what to do in a social situation. | 4.23 | 1.28 | 0.099 | 0.901 | 5.02 | 1.60 | 0.000 | 1.000 | 4.23 | 1.35 | 0.112 | 0.888 | 4.00 | 1.81 | N/A | N/A |
| EMP4 | Judging if something is rude or polite. | 3.67 | 1.46 | 0.943 | 0.057 | 4.58 | 1.70 | 0.008 | 0.992 | 3.98 | 1.39 | 0.540 | 0.460 | 3.41 | 1.80 | N/A | N/A |
| EMP5 | Focusing on one’s own thoughts rather than on what their listener might be thinking. | 4.59 | 1.36 | 0.002 | 0.998 | 4.46 | 1.65 | 0.025 | 0.975 | 3.87 | 1.40 | 0.754 | 0.246 | 4.15 | 1.67 | N/A | N/A |
| EMP6 | Picking up quickly if Someone says one thing but means another. | 3.80 | 1.56 | 0.864 | 0.136 | 4.62 | 1.62 | 0.001 | 0.999 | 4.05 | 1.48 | 0.379 | 0.621 | 4.24 | 1.69 | N/A | N/A |
| EMP7 | Seeing why some things upset people so much. | 3.74 | 1.58 | 0.923 | 0.077 | 4.42 | 1.71 | 0.018 | 0.982 | 3.83 | 1.45 | 0.846 | 0.154 | 4.35 | 1.70 | N/A | N/A |
| EMP8 | Putting oneself in somebody else's shoes. | 3.74 | 1.45 | 0.942 | 0.058 | 4.57 | 1.68 | 0.002 | 0.998 | 3.96 | 1.49 | 0.591 | 0.409 | 3.71 | 1.94 | N/A | N/A |
| EMP9 | Predicting how someone will feel. | 3.88 | 1.64 | 0.734 | 0.266 | 4.46 | 1.72 | 0.011 | 0.989 | 3.99 | 1.64 | 0.528 | 0.472 | 4.17 | 1.78 | N/A | N/A |
| SYS1 | Obtaining specific information about a car’s engine capacity. | 5.21 | 1.14 | 0.000 | 1.000 | 6.06 | 1.00 | 0.000 | 1.000 | 5.42 | 1.35 | 0.000 | 1.000 | 4.41 | 1.70 | N/A | N/A |
| SYS10 | Noticing the details of how a piece of furniture was constructed. | 5.08 | 1.33 | 0.000 | 1.000 | 5.30 | 1.49 | 0.000 | 1.000 | 5.06 | 1.54 | 0.000 | 1.000 | 4.20 | 1.95 | N/A | N/A |
| SYS11 | Learning one’s way around a new city. | 4.62 | 1.14 | 0.000 | 1.000 | 5.53 | 1.38 | 0.000 | 1.000 | 4.75 | 1.31 | 0.000 | 1.000 | 3.62 | 2.10 | N/A | N/A |
| SYS12 | Watching science documentaries on television or reading articles about science and nature. | 4.68 | 1.28 | 0.000 | 1.000 | 4.70 | 1.64 | 0.002 | 0.998 | 4.48 | 1.16 | 0.003 | 0.997 | 4.08 | 2.03 | N/A | N/A |
| SYS13 | Knowing about a stereo’s precise technical features. | 5.06 | 1.09 | 0.000 | 1.000 | 6.09 | 1.09 | 0.000 | 1.000 | 5.00 | 1.19 | 0.000 | 1.000 | 3.72 | 2.12 | N/A | N/A |
| SYS14 | Grasping exactly how odds work in betting. | 4.73 | 1.11 | 0.000 | 1.000 | 5.50 | 1.44 | 0.000 | 1.000 | 4.68 | 1.19 | 0.000 | 1.000 | 3.89 | 1.85 | N/A | N/A |
| SYS15 | Being meticulous when carrying out D.I.Y. | 4.22 | 1.22 | 0.107 | 0.893 | 4.65 | 1.56 | 0.002 | 0.998 | 4.27 | 1.31 | 0.071 | 0.929 | 3.61 | 1.88 | N/A | N/A |
| SYS16 | Being curious about the precise way a building was constructed. | 5.08 | 1.17 | 0.000 | 1.000 | 4.54 | 1.71 | 0.014 | 0.986 | 4.90 | 1.09 | 0.000 | 1.000 | 3.77 | 1.94 | N/A | N/A |
| SYS17 | Understanding information the bank sends on different investment and saving systems. | 4.57 | 1.21 | 0.000 | 1.000 | 6.01 | 1.08 | 0.000 | 1.000 | 4.72 | 1.10 | 0.000 | 1.000 | 3.82 | 2.05 | N/A | N/A |
| SYS18 | Wondering exactly how rail networks of trains are coordinated. | 5.00 | 1.37 | 0.000 | 1.000 | 5.37 | 1.73 | 0.000 | 1.000 | 5.02 | 1.47 | 0.000 | 1.000 | 4.00 | 2.01 | N/A | N/A |
| SYS19 | Looking carefully into the quality of a camera lens. | 4.35 | 1.06 | 0.011 | 0.989 | 5.88 | 1.19 | 0.000 | 1.000 | 4.35 | 1.27 | 0.027 | 0.973 | 3.23 | 2.14 | N/A | N/A |
| SYS2 | Fixing a problem with the electrical wiring in one’s home. | 5.19 | 1.22 | 0.000 | 1.000 | 5.88 | 1.31 | 0.000 | 1.000 | 5.25 | 1.20 | 0.000 | 1.000 | 3.85 | 1.93 | N/A | N/A |
| SYS20 | Being interested in meteorological patterns. | 4.52 | 1.06 | 0.000 | 1.000 | 4.65 | 1.71 | 0.004 | 0.996 | 4.54 | 1.21 | 0.001 | 0.999 | 3.60 | 1.92 | N/A | N/A |
| SYS21 | Thinking about how precisely a mountain was formed. | 4.57 | 0.96 | 0.000 | 1.000 | 5.15 | 1.63 | 0.000 | 1.000 | 4.69 | 1.05 | 0.000 | 1.000 | 3.53 | 2.17 | N/A | N/A |
| SYS22 | Visualizing how the motorways in one’s region link up. | 4.73 | 1.25 | 0.000 | 1.000 | 5.64 | 1.35 | 0.000 | 1.000 | 4.68 | 1.27 | 0.000 | 1.000 | 3.73 | 2.00 | N/A | N/A |
| SYS23 | Thinking about the aerodynamics of a plane. | 4.85 | 1.12 | 0.000 | 1.000 | 5.51 | 1.58 | 0.000 | 1.000 | 4.94 | 1.42 | 0.000 | 1.000 | 3.83 | 2.07 | N/A | N/A |
| SYS24 | Knowing the path a river takes from its source to the sea. | 4.77 | 1.29 | 0.000 | 1.000 | 5.83 | 1.31 | 0.000 | 1.000 | 4.87 | 1.27 | 0.000 | 1.000 | 3.83 | 1.90 | N/A | N/A |
| SYS25 | Understanding how wireless communication works. | 4.77 | 1.09 | 0.000 | 1.000 | 6.08 | 1.29 | 0.000 | 1.000 | 4.80 | 1.19 | 0.000 | 1.000 | 3.51 | 2.04 | N/A | N/A |
| SYS3 | Reading articles or web pages about new technology. | 4.92 | 1.16 | 0.000 | 1.000 | 5.52 | 1.25 | 0.000 | 1.000 | 4.49 | 1.21 | 0.001 | 0.999 | 3.57 | 1.90 | N/A | N/A |
| SYS4 | Enjoying games that involve a high degree of strategy. | 4.69 | 1.16 | 0.000 | 1.000 | 4.57 | 1.79 | 0.013 | 0.987 | 4.88 | 1.29 | 0.000 | 1.000 | 3.78 | 1.97 | N/A | N/A |
| SYS5 | Being fascinated by how machines work. | 5.39 | 1.24 | 0.000 | 1.000 | 4.27 | 1.78 | 0.111 | 0.889 | 5.19 | 1.42 | 0.000 | 1.000 | 4.21 | 1.97 | N/A | N/A |
| SYS6 | Being intrigued by the rules and patterns governing numbers in math. | 4.74 | 1.42 | 0.000 | 1.000 | 4.50 | 1.99 | 0.022 | 0.978 | 4.74 | 1.35 | 0.000 | 1.000 | 4.02 | 1.97 | N/A | N/A |
| SYS7 | Understanding instruction manuals for putting appliances together. | 4.80 | 1.34 | 0.000 | 1.000 | 5.40 | 1.41 | 0.000 | 1.000 | 4.47 | 1.95 | 0.030 | 0.970 | 4.58 | 1.93 | N/A | N/A |
| SYS8 | Knowing exact details about a computer’s hard disk drive capacity and processor speed. | 4.77 | 1.58 | 0.000 | 1.000 | 6.15 | 1.17 | 0.000 | 1.000 | 4.88 | 1.37 | 0.000 | 1.000 | 4.17 | 2.06 | N/A | N/A |
| SYS9 | Reading and understanding maps. | 4.75 | 1.23 | 0.000 | 1.000 | 5.83 | 1.05 | 0.000 | 1.000 | 4.83 | 1.21 | 0.000 | 1.000 | 3.67 | 2.14 | N/A | N/A |

*Note.* MP = Scale midpoint. Tests against midpoint is not provided for Genetic Difference ratings since comparison to scale midpoint had no clear meaning in Study 2.

*Study 2 -* *Descriptives by Coder Gender*

**Table S.3. Mean coder ratings among men and women coders separately, as well as the effect size for the gender difference between men and women coders (Study 2)**

|  | **SQ-Short Activities** | | | **EQ-Short Activities** | | |
| --- | --- | --- | --- | --- | --- | --- |
| **Rating Dimension** | **Men**  ***N* = 99**  *M* (SD) | **Women**  ***N* = 98**  *M* (SD) | **Gender Difference**  (*d, p*) | **Men**  ***N* = 99**  *M* (SD) | **Women**  ***N* = 98**  *M* (SD) | **Gender Difference**  (*d, p*) |
| Estimated Gender Difference | 4.92 (0.27) | 4.67 (0.39) | .76*  *p =* .010 | 4.07  (0.46) | 3.42 (0.30) | 1.69***  *p* < .001 |
| Learned vs. Innate Attributions | 5.50 (0.54) | 5.26 (0.71) | .38  *p* = .187 | 4.66 (0.36) | 4.30 (0.35) | 1.00**  *p =* .002 |
| Gendered Learning Affordances | 4.95 (0.27) | 4.64 (0.35) | 1.01***  *p* < .001 | 4.30 (0.41) | 3.65 (0.40) | 1.58***  *p* < .001 |
| Genetic Differences | 3.90 (0.40) | 3.80 (0.36) | .26  *p* = .369 | 4.18 (0.27) | 3.89 (0.42) | .81*  *p* = .011 |

*Note.* Below Midpoint = Women Higher, More Innate; Above Midpoint = Men Higher, More Learned. **p* < .05. ***p* < .01. ****p* < .001. There were no non-binary coders in Study 2

*Study 2 - Effects by Coder Gender and for Overall Sample*

**Table S.4. Effects predicting the magnitude of the gender difference on each item from mean coder ratings among men and women coders separately (Study 2)**

|  | Effects for Men  (*N =* 99) | | | Effects for Women  (*N =* 98) | | |
| --- | --- | --- | --- | --- | --- | --- |
| Rating | Main Effect  (𝛽) | Rating $\times$ Type  Interaction? | Simple Slope  (𝛽) | Main Effect  (𝛽) | Rating $\times$ Type  Interaction? | Simple Slope  (𝛽) |
| Estimated Gender Difference | .37***  *p* < .001 | Yes | Sys: .60**  Emp: .14 | .52***  *p* < .001 | No | --- |
| Learned vs. Innate Attributions | .10  *p* = .341 | No | --- | .29*  *p* = .010 | No | --- |
| Gendered Learning Affordances | .24*  *p* = .017 | No | --- | .46***  *p* < .001 | No | --- |
| Genetic Difference | -.05  *p* = .538 | No | --- | .01  *p* = .887 | No | --- |

|  | Overall Effect for Reference  (*N =* 199) | | |
| --- | --- | --- | --- |
| Rating | Main Effect  (𝛽) | Rating $\times$ Type  Interaction? | Simple Slopes  (Reported for All Outcomes)  (𝛽) |
| Estimated Gender Difference | .64***  *p* < .001 | No | Emp: .54**  Sys: .74*** |
| Learned vs. Innate Attributions | .31*  *p* = .022 | No | Emp: .52*  Sys: .10 |
| Gendered Learning Affordances | .47***  *p* < .001 | No | Emp: .45**  Sys: .49*** |
| Genetic Difference | -.02  *p* = .804 | No | Emp: -.15  Sys: .12 |

**Study 3**

*Study 3 -* *List of Measures*

Below is the full list of variables included in Study 3 in the order they were presented to participants. Variables analyzed and reported in the main text are denoted with an asterisk.

- Learned vs. Innate Attributions*
- Estimated Gender Difference*
- The order of the following variables was counterbalanced:
  - Gendered Learning Affordances*
  - Genetic Differences*
- Fixed vs. Growth Mindset
- Gender*
- Age*
- Year Received PhD
- Current Position
- Area of Expertise
- Ethnic/Racial Background*
- Political Orientation*
- SES*
- Honesty Check*

*Study 3 -* *Items for Measures*

**SQ Activities**

- 1. Obtaining specific information about a car’s engine capacity.
- 2. Fixing a problem with the electrical wiring in one’s home.
- 3. Reading articles or web pages about new technology.
- 4. Enjoying games that involve a high degree of strategy.
- 5. Being fascinated by how machines work.
- 6. Being intrigued by the rules and patterns governing numbers in math.
- 7. Understanding instruction manuals for putting appliances together.
- 8. Knowing exact details about a computer’s hard disk drive capacity and processor speed.
- 9. Reading and understanding maps.
- 10. Noticing the details of how a piece of furniture was constructed.
- 11. Learning one’s way around a new city.
- 12. Watching science documentaries on television or reading articles about science and nature.
- 13. Knowing about a stereo’s precise technical features.
- 14. Grasping exactly how odds work in betting.
- 15. Being meticulous when carrying out D.I.Y.
- 16. Being curious about the precise way a building was constructed.
- 17. Understanding information the bank sends on different investment and saving systems.
- 18. Wondering exactly how rail networks of trains are coordinated.
- 19. Looking carefully into the quality of a camera lens.
- 20. Being interested in meteorological patterns.
- 21. Thinking about how precisely a mountain was formed.
- 22. Visualizing how the motorways in one’s region link up.
- 23. Thinking about the aerodynamics of a plane.
- 24. Knowing the path a river takes from its source to the sea.
- 25. Understanding how wireless communication works.

**EQ Activities**

*(R) =* Reverse-scored

Although not preregistered, we reverse-scored EQ Activities 5, 12, and 17 for directional ratings of the gender difference, gendered learning opportunities, and genetic advantage, since these activities are negatively worded (i.e., higher values corresponded with less empathizing). Not reverse-scoring these activities only changes 2 results: for empathizing items, (1) the relationship between estimate of gender difference and the size of the gender difference becomes non-significant, and (2) the relationship between gendered learning affordances and the size of the gender difference becomes non-significant.

- 1. Telling if someone else wants to enter a conversation.
- 2. Caring for other people.
- 3. Knowing what to do in a social situation.
- 4. Judging if something is rude or polite.
- 5. Focusing on one’s own thoughts rather than on what their listener might be thinking. *(R)*
- 6. Picking up quickly if someone says one thing but means another.
- 7. Seeing why some things upset people so much.
- 8. Putting oneself in somebody else's shoes.
- 9. Predicting how someone will feel.
- 10. Spotting when someone in a group is feeling awkward or uncomfortable.
- 11. Seeing why someone should have felt offended by a remark.
- 12. Finding social situations confusing. *(R)*
- 13. Understanding how other people are feeling and what they are thinking.
- 14. Telling if someone else is interested or bored with what one is saying.
- 15. Being very understanding.
- 16. Sensing if one is intruding.
- 17. Being insensitive. *(R)*
- 18. Tuning into how someone else feels.
- 19. Working out what another person might want to talk about.
- 20. Telling if someone is masking their true emotion.
- 21. Predicting what someone will do.
- 22. Getting emotionally involved with a friend's problems.

**Honesty Check**

Given that we are only contacting a few experts in psychology to participate in our survey, individual data quality is extremely important to our study. We would like to give you the opportunity to withdraw your response if you feel you did not answer these questions to your full abilities.

Should we include your response in our dataset?

Please note: Your response to this question has NO bearing whatsoever on your participation nor eligibility to receive a summary report of the study's findings.

- **YES**, I answered all questions to the best of my ability. **Please use my data.**
- **NO**, I did not answer all questions to the best of my ability. **Do not use my data.**

*Study 3 – Recruitment Strategy*

Our preregistered goal was to collect data from *N =* 140 experts from 7 subdisciplines of psychology: (1) Social/Personality, (2) Developmental, (3) Neuro/Cognitive, (4) Clinical, (5) General, (6) Evolutionary, and (7) Gender (*N =* 20 per discipline, 10 men and 10 women per discipline). Although 2 to 3 coders are recommended for typical interrater reliability estimates (Gisev et al., 2013; Lavrakas, 2008), given the subjective nature of our ratings and our wisdom of the crowds approach, we opted to collect *N =* 10 per cross-section of gender and discipline. Assuming a 20% response rate, we originally planned to contact *N =* 700 experts (*N =* 100 per discipline), but actually sent invitations to only 600, as explained below.

We randomly sampled experts from editorial boards of the most influential journals in each subdiscipline of psychology. To start, we generated a list of top 20 journals from SCImago Journal & Country Rank database. To identify influential journals, although an imperfect proxy, we sorted journals by H index for 2019 and subdiscipline. The initial list was reviewed by the research team and journals that did not directly correspond to the subdiscipline in question (e.g., clinical journals in developmental psychology) were removed. We then shared our initial list with experts from each subdiscipline and adjusted our list according to their feedback (i.e., if there were any journals that did not belong on the list, or if there were key journals missing from the list). Next, we cross-checked our revised journal list against journals affiliated with two major psychological organizations: the American Psychological Association and the Association for Psychological Science and added any missing journals in each subcategory. Finally, in order to triangulate on another common journal metric, we sorted our entire list by impact factor and selected the 10 journals with the highest impact factor from each subdiscipline. In cases where journals appeared twice on different lists, we assigned the journal to the discipline it was most related to. Our final list included *N =* 60 journals.^^[[1]](#footnote-1)^^

Next, as preregistered, we compiled a contact list based on publicly available contact information listed on the editorial board of each journal’s page. Our research team recorded each expert’s name, institution, editorial board position, email address, gender (based on pronouns or other information, when possible), and whether they were a psychologist (verified through their professional webpage or faculty website). Entries were filtered for eligibility (is a psychologist, email located, is an editorial board member) and cross-checked for duplicate email addresses. This resulted in a contact list of *N =* 2374 eligible participants (1081 women, 1293 men). From this list, we randomly sampled *N =* 100 experts (50 women, 50 men) from each subdiscipline. For evolutionary and gender psychology, given that we had a lower number of journals overall, and given there were not enough eligible women in evolutionary psychology, we lowered our sampling to *N =* 50 experts (25 women, 25 men) for these two subdisciplines only (this sampling decision was unforeseen, and our final recruitment target of 600 experts deviated from our preregistered target of 700).

A personalized invitation to participate in the survey was sent out to *N =* 600 experts. A reminder was sent out one week later. *N =* 8 email addresses were unable to receive email, and *N =* 4 participants were unable to complete the survey. To ensure we met our target sample size, we substituted these contacts with *N =* 12 experts randomly sampled from the same discipline and gender as the original participants. In total, we contacted *N =* 612 experts.

*Study 3 – Item-Level Descriptive Statistics*

**Table S.5. Item-level information for coder ratings for each measure and the p-value for t-tests comparing ratings to scale midpoint (Study 3)**

| Variable | Item Text | Gender Differences | | | | Innate vs. Learned Attributions | | | | Gendered Learning Affordances | | | | Genetic Differences | | | |
| --- | --- | --- | --- | --- | --- | --- | --- | --- | --- | --- | --- | --- | --- | --- | --- | --- | --- |
|  |  | Mean | SD | Above MP  (*p*) | Below MP  (*p*) | Mean | SD | Above MP  (*p*) | Below MP  (*p*) | Mean | SD | Above MP  (*p*) | Below MP  (*p*) | Mean | SD | Above MP  (*p*) | Below MP  (*p*) |
| EMP1 | Telling if someone else wants to enter a conversation. | 3.29 | 0.86 | 1.000 | 0.000 | 4.36 | 1.47 | 0.045 | 0.955 | 3.69 | 1.10 | 0.976 | 0.024 | 3.67 | 0.77 | 0.998 | 0.002 |
| EMP10 | Spotting when someone in a group is feeling awkward or uncomfortable. | 3.24 | 0.74 | 1.000 | 0.000 | 3.91 | 1.49 | 0.707 | 0.293 | 3.42 | 0.84 | 1.000 | 0.000 | 3.76 | 0.52 | 1.000 | 0.000 |
| EMP11 | Seeing why someone should have felt offended by a remark. | 3.20 | 0.74 | 1.000 | 0.000 | 4.47 | 1.50 | 0.004 | 0.996 | 3.35 | 0.95 | 1.000 | 0.000 | 3.72 | 0.61 | 1.000 | 0.000 |
| EMP12 | Finding social situations confusing. | 4.15 | 0.60 | 0.030 | 0.970 | 3.65 | 1.49 | 0.963 | 0.037 | 3.74 | 0.70 | 0.997 | 0.003 | 3.89 | 0.52 | 0.955 | 0.045 |
| EMP13 | Understanding how other people are feeling and what they are thinking. | 3.34 | 0.67 | 1.000 | 0.000 | 4.08 | 1.51 | 0.322 | 0.678 | 3.22 | 0.83 | 1.000 | 0.000 | 3.70 | 0.62 | 1.000 | 0.000 |
| EMP14 | Telling if someone else is interested or bored with what one is saying. | 3.38 | 0.68 | 1.000 | 0.000 | 4.35 | 1.36 | 0.033 | 0.967 | 3.45 | 0.96 | 1.000 | 0.000 | 3.82 | 0.43 | 0.998 | 0.002 |
| EMP15 | Being very understanding. | 3.22 | 0.87 | 1.000 | 0.000 | 4.03 | 1.44 | 0.437 | 0.563 | 3.26 | 0.93 | 1.000 | 0.000 | 3.61 | 0.71 | 1.000 | 0.000 |
| EMP16 | Sensing if one is intruding. | 3.49 | 0.82 | 1.000 | 0.000 | 4.49 | 1.35 | 0.001 | 0.999 | 3.49 | 0.84 | 1.000 | 0.000 | 3.76 | 0.51 | 1.000 | 0.000 |
| EMP17 | Being insensitive. | 4.73 | 0.70 | 0.000 | 1.000 | 3.71 | 1.38 | 0.971 | 0.029 | 4.23 | 1.01 | 0.020 | 0.980 | 4.11 | 0.66 | 0.065 | 0.935 |
| EMP18 | Tuning into how Someone else feels. | 3.18 | 0.75 | 1.000 | 0.000 | 3.92 | 1.38 | 0.686 | 0.314 | 3.21 | 0.87 | 1.000 | 0.000 | 3.49 | 0.75 | 1.000 | 0.000 |
| EMP19 | Working out what another person might want to talk about. | 3.62 | 0.64 | 1.000 | 0.000 | 4.81 | 1.43 | 0.000 | 1.000 | 3.43 | 0.78 | 1.000 | 0.000 | 3.86 | 0.45 | 0.983 | 0.017 |
| EMP2 | Caring for other people. | 3.09 | 0.73 | 1.000 | 0.000 | 3.94 | 1.46 | 0.648 | 0.352 | 3.11 | 1.20 | 1.000 | 0.000 | 3.63 | 0.64 | 1.000 | 0.000 |
| EMP20 | Telling if someone is masking their true emotion. | 3.48 | 0.73 | 1.000 | 0.000 | 4.48 | 1.55 | 0.015 | 0.985 | 3.42 | 0.91 | 1.000 | 0.000 | 3.63 | 0.71 | 1.000 | 0.000 |
| EMP21 | Predicting what Someone will do. | 3.90 | 0.45 | 0.933 | 0.067 | 4.73 | 1.46 | 0.000 | 1.000 | 3.94 | 0.51 | 0.795 | 0.205 | 3.76 | 0.55 | 0.998 | 0.002 |
| EMP22 | Getting emotionally involved with a friend's problems. | 2.97 | 0.77 | 1.000 | 0.000 | 3.94 | 1.39 | 0.659 | 0.341 | 2.92 | 0.95 | 1.000 | 0.000 | 3.63 | 0.69 | 1.000 | 0.000 |
| EMP3 | Knowing what to do in a social situation. | 3.50 | 0.57 | 1.000 | 0.000 | 4.93 | 1.44 | 0.000 | 1.000 | 3.38 | 0.89 | 1.000 | 0.000 | 3.70 | 0.63 | 1.000 | 0.000 |
| EMP4 | Judging if something is rude or polite. | 3.40 | 0.68 | 1.000 | 0.000 | 4.95 | 1.34 | 0.000 | 1.000 | 3.47 | 0.81 | 1.000 | 0.000 | 3.75 | 0.64 | 0.998 | 0.002 |
| EMP5 | Focusing on one’s own thoughts rather than on what their listener might be thinking. | 4.15 | 0.96 | 0.131 | 0.869 | 4.06 | 1.22 | 0.368 | 0.632 | 3.76 | 0.85 | 0.979 | 0.021 | 3.93 | 0.54 | 0.839 | 0.161 |
| EMP6 | Picking up quickly if Someone says one thing but means another. | 3.61 | 0.76 | 1.000 | 0.000 | 4.18 | 1.55 | 0.196 | 0.804 | 3.56 | 0.95 | 0.999 | 0.001 | 3.76 | 0.64 | 0.996 | 0.004 |
| EMP7 | Seeing why some things upset people so much. | 3.14 | 0.71 | 1.000 | 0.000 | 4.36 | 1.40 | 0.014 | 0.986 | 3.23 | 0.90 | 1.000 | 0.000 | 3.66 | 0.60 | 1.000 | 0.000 |
| EMP8 | Putting oneself in Somebody else's shoes. | 3.27 | 0.65 | 1.000 | 0.000 | 3.90 | 1.42 | 0.736 | 0.264 | 3.24 | 0.96 | 1.000 | 0.000 | 3.56 | 0.66 | 1.000 | 0.000 |
| EMP9 | Predicting how someone will feel. | 3.14 | 0.69 | 1.000 | 0.000 | 4.38 | 1.51 | 0.015 | 0.985 | 3.19 | 0.93 | 1.000 | 0.000 | 3.67 | 0.62 | 1.000 | 0.000 |
| SYS1 | Obtaining specific information about a car’s engine capacity. | 4.85 | 0.97 | 0.000 | 1.000 | 6.15 | 1.03 | 0.000 | 1.000 | 5.25 | 1.05 | 0.000 | 1.000 | 4.27 | 0.56 | 0.001 | 0.999 |
| SYS10 | Noticing the details of how a piece of furniture was constructed. | 4.48 | 0.73 | 0.000 | 1.000 | 5.28 | 1.32 | 0.000 | 1.000 | 4.54 | 0.92 | 0.000 | 1.000 | 4.10 | 0.57 | 0.114 | 0.886 |
| SYS11 | Learning one’s way around a new city. | 4.46 | 0.65 | 0.000 | 1.000 | 4.64 | 1.34 | 0.001 | 0.999 | 4.38 | 0.78 | 0.001 | 0.999 | 4.26 | 0.63 | 0.003 | 0.997 |
| SYS12 | Watching science documentaries on television or reading articles about science and nature. | 4.27 | 0.52 | 0.000 | 1.000 | 4.92 | 1.39 | 0.000 | 1.000 | 4.36 | 0.55 | 0.000 | 1.000 | 4.05 | 0.22 | 0.042 | 0.958 |
| SYS13 | Knowing about a stereo’s precise technical features. | 5.11 | 0.81 | 0.000 | 1.000 | 6.09 | 0.96 | 0.000 | 1.000 | 5.12 | 0.91 | 0.000 | 1.000 | 4.14 | 0.41 | 0.016 | 0.984 |
| SYS14 | Grasping exactly how odds work in betting. | 4.35 | 0.68 | 0.000 | 1.000 | 5.40 | 1.39 | 0.000 | 1.000 | 4.60 | 0.70 | 0.000 | 1.000 | 4.12 | 0.33 | 0.006 | 0.994 |
| SYS15 | Being meticulous when carrying out D.I.Y. | 4.00 | 0.58 | 0.500 | 0.500 | 4.30 | 1.45 | 0.069 | 0.931 | 4.28 | 0.69 | 0.002 | 0.998 | 4.00 | 0.51 | 0.500 | 0.500 |
| SYS16 | Being curious about the precise way a building was constructed. | 4.49 | 0.69 | 0.000 | 1.000 | 4.38 | 1.47 | 0.030 | 0.970 | 4.69 | 0.88 | 0.000 | 1.000 | 4.11 | 0.50 | 0.055 | 0.945 |
| SYS17 | Understanding information the bank sends on different investment and saving systems. | 4.16 | 0.55 | 0.022 | 0.978 | 5.92 | 1.12 | 0.000 | 1.000 | 4.50 | 0.86 | 0.000 | 1.000 | 4.06 | 0.31 | 0.091 | 0.909 |
| SYS18 | Wondering exactly how rail networks of trains are coordinated. | 4.87 | 0.84 | 0.000 | 1.000 | 4.79 | 1.64 | 0.000 | 1.000 | 4.70 | 0.84 | 0.000 | 1.000 | 4.19 | 0.62 | 0.016 | 0.984 |
| SYS19 | Looking carefully into the quality of a camera lens. | 4.35 | 0.73 | 0.000 | 1.000 | 5.78 | 1.13 | 0.000 | 1.000 | 4.40 | 0.60 | 0.000 | 1.000 | 4.11 | 0.42 | 0.029 | 0.971 |
| SYS2 | Fixing a problem with the electrical wiring in one’s home. | 4.90 | 0.82 | 0.000 | 1.000 | 5.88 | 1.06 | 0.000 | 1.000 | 5.23 | 0.96 | 0.000 | 1.000 | 4.21 | 0.54 | 0.003 | 0.997 |
| SYS20 | Being interested in meteorological patterns. | 4.24 | 0.55 | 0.002 | 0.998 | 4.94 | 1.59 | 0.000 | 1.000 | 4.26 | 0.53 | 0.001 | 0.999 | 4.14 | 0.45 | 0.017 | 0.983 |
| SYS21 | Thinking about how precisely a mountain was formed. | 4.21 | 0.56 | 0.003 | 0.997 | 5.30 | 1.39 | 0.000 | 1.000 | 4.48 | 0.69 | 0.000 | 1.000 | 4.07 | 0.33 | 0.051 | 0.949 |
| SYS22 | Visualizing how the motorways in one’s region link up. | 4.54 | 0.73 | 0.000 | 1.000 | 4.30 | 1.46 | 0.076 | 0.924 | 4.55 | 0.79 | 0.000 | 1.000 | 4.20 | 0.46 | 0.001 | 0.999 |
| SYS23 | Thinking about the aerodynamics of a plane. | 4.73 | 0.65 | 0.000 | 1.000 | 5.71 | 1.14 | 0.000 | 1.000 | 4.80 | 0.90 | 0.000 | 1.000 | 4.11 | 0.49 | 0.067 | 0.933 |
| SYS24 | Knowing the path a river takes from its source to the sea. | 4.20 | 0.45 | 0.001 | 0.999 | 6.00 | 1.03 | 0.000 | 1.000 | 4.19 | 0.59 | 0.012 | 0.988 | 3.98 | 0.31 | 0.670 | 0.330 |
| SYS25 | Understanding how wireless communication works. | 4.52 | 0.79 | 0.000 | 1.000 | 5.54 | 1.46 | 0.000 | 1.000 | 4.48 | 0.82 | 0.000 | 1.000 | 4.08 | 0.58 | 0.161 | 0.839 |
| SYS3 | Reading articles or web pages about new technology. | 4.69 | 0.84 | 0.000 | 1.000 | 5.29 | 1.30 | 0.000 | 1.000 | 4.84 | 0.81 | 0.000 | 1.000 | 4.20 | 0.53 | 0.005 | 0.995 |
| SYS4 | Enjoying games that involve a high degree of strategy. | 4.51 | 0.74 | 0.000 | 1.000 | 4.29 | 1.41 | 0.066 | 0.934 | 4.78 | 0.74 | 0.000 | 1.000 | 4.16 | 0.50 | 0.009 | 0.991 |
| SYS5 | Being fascinated by how machines work. | 5.06 | 0.85 | 0.000 | 1.000 | 4.17 | 1.52 | 0.207 | 0.793 | 5.20 | 1.00 | 0.000 | 1.000 | 4.29 | 0.73 | 0.003 | 0.997 |
| SYS6 | Being intrigued by the rules and patterns governing numbers in math. | 4.68 | 0.83 | 0.000 | 1.000 | 3.57 | 1.48 | 0.983 | 0.017 | 4.65 | 0.99 | 0.000 | 1.000 | 4.27 | 0.56 | 0.000 | 1.000 |
| SYS7 | Understanding instruction manuals for putting appliances together. | 4.42 | 0.83 | 0.000 | 1.000 | 5.11 | 1.20 | 0.000 | 1.000 | 4.80 | 0.89 | 0.000 | 1.000 | 4.31 | 0.66 | 0.001 | 0.999 |
| SYS8 | Knowing exact details about a computer’s hard disk drive capacity and processor speed. | 4.78 | 0.86 | 0.000 | 1.000 | 6.12 | 1.05 | 0.000 | 1.000 | 5.00 | 0.89 | 0.000 | 1.000 | 4.18 | 0.48 | 0.006 | 0.994 |
| SYS9 | Reading and understanding maps. | 4.44 | 0.74 | 0.000 | 1.000 | 5.25 | 1.49 | 0.000 | 1.000 | 4.35 | 0.81 | 0.002 | 0.998 | 4.23 | 0.63 | 0.007 | 0.993 |

*Note.* MP = Scale midpoint.

*Study 3 -* *Descriptives by Coder Gender*

**Table S.6. Mean coder ratings among men and women coders separately, as well as the effect size for the gender difference between men and women coders (Study 3)**

|  | **SQ-Short Activities** | | | **EQ-Short Activities** | | |
| --- | --- | --- | --- | --- | --- | --- |
| **Rating Dimension** | **Men**  ***N* = 52**  *M* (SD) | **Women**  ***N* = 64**  *M* (SD) | **Gender Difference**  (*d, p*) | **Men**  ***N* = 52**  *M* (SD) | **Women**  ***N* = 64**  *M* (SD) | **Gender Difference**  (*d, p*) |
| Estimated Gender Difference | 4.60 (0.33) | 4.44 (0.26) | .53  *p =* .067 | 3.36 (0.27) | 3.41 (0.26) | .21  *p =* .500 |
| Learned vs. Innate Attributions | 5.18 (0.69) | 5.15 (0.80) | .04  *p =* .878 | 4.26 (0.35) | 4.25 (0.46) | .02  *p =* .956 |
| Gendered Learning Affordances | 4.59 (0.34) | 4.75 (0.30) | .51  *p =* .077 | 3.61 (0.27) | 3.29 (0.45) | .86**  *p =* .007 |
| Genetic Advantage | 4.18 (0.12) | 4.13 (0.09) | .48  *p =* .095 | 3.65 (0.22) | 3.84 (0.09) | 1.14***  *p <* .001 |

*Note.* Below Midpoint = Women Higher, More Innate; Above Midpoint = Men Higher, More Learned. **p* < .05. ***p* < .01. ****p* < .001.

*Study 3 -* *Descriptives by Coder Subdiscipline*

Below are mean coder ratings among each subdiscipline separately. A one-way ANOVA comparing ratings by subdiscipline revealed significant differences on all variables across subdiscipline. Notably, gender and evolutionary scholars tended to be most extreme in their perceptions of the etiology of gender differences, with ratings that were more often significantly different than those from other subdisciplines. For example, evolutionary scholars were significantly different than all other subdisciplines in their belief that gender differences in systemizing and empathizing are due to sex-linked genetic advantages. Gender scholars were significantly higher than most subdisciplines in believing that empathizing and systemizing skills were more learnable than innate.

Table S.7. Mean coder ratings by coder subdiscipline (Study 3)

| **SQ-Short Activities** | | | | | | | |
| --- | --- | --- | --- | --- | --- | --- | --- |
| **Rating Dimension** | **Clin**  ***N* = 12**  *M* (SD) | **Dev**  ***N* = 16**  *M* (SD) | **Evo**  ***N* = 15**  *M* (SD) | **Gend**  ***N* = 7**  *M* (SD) | **Genr**  ***N* = 12**  *M* (SD) | **Neuro**  ***N* = 15**  *M* (SD) | **S/P**  ***N* = 30**  *M* (SD) |
|  |  |  |  |  |  |  |  |
| Estimated Gender Difference | 4.40_b_ (0.39) | 4.46_b_ (0.27) | 4.85_a_ (0.47) | 4.43_b_ (0.36) | 4.60_ab_  (0.46) | 4.43_b_ (0.32) | 4.56_ab_ (0.35) |
|  |  | ** | ** | ** | ** |  |  |
|  |  |  | ***  ** |  | ***  *** | * |  |
| Learned vs. Innate Attributions | 4.68_def_ (1.18) | 5.42_bc_ (0.67) | 4.27_f_ (0.92) | 6.33_a_ (0.57) | 5.55_ab_ (0.73) | 5.30_bcd_ (0.94) | 5.04_bcde_ (0.84) |
|  |  | * | ***  *** | *** |  | *** |  |
|  |  |  | ** |  |  |  |  |
| Gendered Learning Affordances | 4.37_a_ (0.57) | 4.93_b_ (0.61) | 4.83_b_ (0.36) | 4.99_b_ (0.52) | 4.59_ab_ (0.45) | 4.66_ab_ (0.46) | 4.61_ab_ (0.31) |
|  |  | ***  * |  |  |  |  |  |
|  |  |  | ** | ***  *** |  |  |  |
| Genetic Advantage | 3.88_a_ (0.24) | 4.15_bc_ (0.16) | 4.53_e_ (0.37) | 4.01_ab_ (0.05) | 4.20_cd_ (0.25) | 4.13_bc_ (0.11) | 4.13_bc_ (0.13) |
|  |  | ***  *** | ***  *** | *** | *  ***  *** |  |  |

*Note.* **p* < .05. ***p* < .01. ****p* < .001. Clin = Clinical, Dev = Developmental, Evo = Evolutionary, Gend = Gender, Genr = General, Neuro = Neuroscience, S/P = Social/Personality. Within each row, means not sharing the same subscript differ significantly using a Tukey HSD test for multiple comparisons.

| **EQ-Short Activities** | | | | | | | |
| --- | --- | --- | --- | --- | --- | --- | --- |
| **Rating Dimension** | **Clin**  ***N* = 12**  *M* (SD) | **Dev**  ***N* = 16**  *M* (SD) | **Evo**  ***N* = 15**  *M* (SD) | **Gend**  ***N* = 7**  *M* (SD) | **Genr**  ***N* = 12**  *M* (SD) | **Neuro**  ***N* = 15**  *M* (SD) | **S/P**  ***N* = 30**  *M* (SD) |
|  |  |  |  |  |  |  |  |
| Estimated Gender Difference | 3.53_a_ (0.29) | 3.41_ab_ (0.35) | 3.14_b_ (0.43) | 3.31_ab_ (0.29) | 3.23_ab_ (0.38) | 3.43_ab_ (0.35) | 3.48_a_ (0.24) |
|  |  | ** |  |  | * |  |  |
|  |  |  | ***  *** | *** | ***  *** | *** |  |
| Learned vs. Innate Attributions | 3.58_ef_ (0.37) | 4.59_b_ (0.50) | 3.22_f_ (0.48) | 5.44_a_ (0.97) | 4.55_bc_ (0.45) | 4.00_de_ (0.62) | 4.51_bcd_ (0.45) |
|  |  | ***  *** | ***  *** | ***  * |  | * | *** |
|  |  |  |  |  |  |  |  |
| Gendered Learning Affordances | 3.51_ab_ (0.46) | 3.34_ab_ (0.56) | 3.40_ab_ (0.47) | 3.04_a_ (0.89) | 3.52_b_ (0.45) | 3.71_b_ (0.41) | 3.53_b_ (0.23) |
|  |  |  |  |  | **  * |  |  |
|  |  |  |  |  |  |  |  |
| Genetic Advantage | 3.78_b_ (0.30) | 3.82_b_ (0.15) | 3.24_a_ (0.44) | 3.97_b_ (0.08) | 3.71_b_ (0.20) | 3.77_b_ (0.27) | 3.84_b_ (0.09) |
|  |  | *** | *** | ***  *** | *  *** |  |  |

*Note.* **p* < .05. ***p* < .01. ****p* < .001. Clin = Clinical, Dev = Developmental, Evo = Evolutionary, Gend = Gender, Genr = General, Neuro = Neuroscience, S/P = Social/Personality. Within each row, means not sharing the same subscript differ significantly using a Tukey HSD test for multiple comparisons.

***

*Study 3 -* *Effects by Coder Gender and for Overall Sample*

**Table S.8. Effects predicting the magnitude of the gender difference on each item from mean coder ratings, among men and women coders separately (Study 3)**

|  | Effects for Men  (*N =* 52) | | | Effects for Women  (*N =* 64) | | |
| --- | --- | --- | --- | --- | --- | --- |
| Rating | Main Effect  (𝛽) | Rating $\times$ Type  Interaction? | Simple Slope  (𝛽) | Main Effect  (𝛽) | Rating $\times$ Type  Interaction? | Simple Slope  (𝛽) |
| Estimated Gender Difference | .68***  *p* < .001 | No | --- | .60***  *p* < .001 | No | --- |
| Learned vs. Innate Attributions | .18  *p* = .100 | No | --- | .21*  *p* = .031 | No | --- |
| Gendered Learning Affordances | .53***  *p* < .001 | No | --- | .61***  *p* < .001 | Yes | Sys: .92***  Emp: .29 |
| Genetic Advantage | .52***  *p* < .001 | Yes | Sys: .89***  Emp: .15 | .18  *p* = .158 | No | --- |

|  | Overall Effect for Reference  (*N =* 116) | | |
| --- | --- | --- | --- |
| Rating | Main Effect  (𝛽) | Rating $\times$ Type  Interaction? | Simple Slopes  (Reported for All Outcomes)  (𝛽) |
| Estimated Gender Difference | .71***  *p* < .001 | No | Emp: .62**  Sys: .81*** |
| Learned vs. Innate Attributions | .20  *p* = .057 | No | Emp: .39*  Sys: .01 |
| Gendered Learning Affordances | .58***  *p* < .001 | Yes | Emp: .35*  Sys: .80*** |
| Genetic Advantage | .51  *p* < .001 | Yes | Emp: .21  Sys: .81*** |

*Study 3 - Effects by Coder Subdiscipline*

Below are effects predicting the magnitude of the gender difference on each item from mean coder ratings among each coder subdiscipline separately, compared to the overall effect for all expert ratings as reported in the main text. In general, social-personality experts tended to have the most accurate predictions, however, the ability to significantly predict the observed gender differences across items and the predictive effect of gender learning affordances on these differences replicated in each of the seven subdisciplines.

**Table S.9. Standardized betas predicting gender differences in target sample by subdiscipline (Study 3)**

| Clin  (*N =* 12) | Dev  (*N =* 16) | Evo  (*N =* 15) | Gend  (*N =* 7) | Genr  (*N =* 12) | Neuro  (*N =* 15) | S/P  (*N =* 30) | **Overall**  **(*N =* 116)** |
| --- | --- | --- | --- | --- | --- | --- | --- |
| Predictor = Estimated Gender Differences | | | | | | | |
| .31**  *p* = .005 | .47***  *p* < .001 | .58***  *p* < .001 | .36**  *p* = .006 | .50***  *p* < .001 | .38***  *p* < .001 | .58***  *p* < .001 | **.71*****  *p* < .001 |
| Predictor = Learned versus Innate Attributions | | | | | | | |
| .18  *p* = .218 | .14  *p* = .113 | .16  *p* = .136 | .08  *p* = .327 | .00  *p* = .994 | .14  *p* = .146 | .21*  *p* = .019 | **.20**  *p* = .057 |
| Predictor = Gendered Learning Affordances | | | | | | | |
| .24**  *p* = .005 | .35**  *p* = .001 | .30*  *p* = .029 | .41***  *p* < .001 | .33***  *p* < .001 | .29**  *p* = .003 | .69***  *p* < .001 | **.58*****  *p* < .001 |
| Predictor = Gendered Genetic Advantages | | | | | | | |
| .09  *p* = .196 | .23*  *p* = .018 | .23  *p* = .068 | .12  *p* = .112 | .08  *p* = .444 | .17  *p* = .170 | .31**  *p* = .007 | **.51*****  *p* < .001 |

*Note.* **p* < .05. ***p* < .01. ****p* < .001. Clin = Clinical, Dev = Developmental, Evo = Evolutionary, Gend = Gender, Genr = General, Neuro = Neuroscience, S/P = Social/Personality

**Studies 2-3**

*Studies 2-3 – Effects Modeled in MLM*

The following tables compare effect sizes modeled in an item-level GLM (as reported in the main text) and MLM framework. MLM analyses predict the size of the gender difference (Study 1) from coder ratings (Studies 2-3), with ratings grand mean centered and nested within coder and subdiscipline (Study 3 only). Since prior models specifying random slopes failed to converge, MLM models specify random intercepts and fixed slopes. MLM analyses are conducted using the R package lmer4 (version 1.1-21; Bates et al., 2015).

While the magnitude of effects generally tracked across GLM and MLM analyses (i.e., in the competing predictor analysis, the effect size for genetic advantage was approximately half the size of the effect of gendered learning affordances in both GLM and MLM), in two cases the MLM analysis was significant where the GLM analysis was not significant. We suspect this is due to the higher sample size for the MLM data (*N=* 2,975 in Study 2, *N=* 2,784 in Study 3).

**Table S.10. Single predictor analyses comparing GLM and MLM estimates (Studies 2 and 3)**

|  | **Study 2** | | **Study 3** | |
| --- | --- | --- | --- | --- |
|  | GLM  (𝛽) | MLM  (*b*) | GLM  (𝛽) | MLM  (*b*) |
| Estimated Gender Difference | .64***  *p <* .001 | .02***  *p <* .001 | .71***  *p <* .001 | .06***  *p <* .001 |
| Learned vs. Innate Attributions | .31*  *p* = .022 | .008***  *p <* .001 | .20  *p* = .057 | .008***  *p <* .001 |
| Gendered Learning Affordances | .47***  *p <* .001 | .02***  *p <* .001 | .58***  *p <* .001 | .05***  *p <* .001 |
| Genetic Differences/Advantage | .02  *p* = .804 | -.001  *p* = .594 | .51***  *p <* .001 | .03***  *p <* .001 |
|  | ***N*** *=* 47 | ***N*** *=* 2,975 | ***N*** *=* 47 | ***N*** *=* 2,784 |

**Table S.11. Competing predictor analysis comparing GLM and MLM estimates (Study 3)**

|  | **Study 3** | |
| --- | --- | --- |
|  | GLM  (𝛽) | MLM  (*b*) |
| Gendered Learning Affordances | .57**  *p* = .003 | .07***  *p* < .001 |
| Genetic  Advantage | .25  *p* = .158 | .03*  *p* = .013 |
|  | ***N*** *=* 47 | ***N*** *=* 2,784 |

1. Because Gender and Evolutionary Psychology contained fewer journals overall, we opted to sample *N =* 5 journals from each of these specialized but highly relevant categories. All other subdisciplines contained *N =* 10 journals per category. [↑](#footnote-ref-1)
